# Exclusive photorelease of signalling lipids at the plasma membrane

**DOI:** 10.1038/ncomms10056

**Published:** 2015-12-21

**Authors:** André Nadler, Dmytro A. Yushchenko, Rainer Müller, Frank Stein, Suihan Feng, Christophe Mulle, Mario Carta, Carsten Schultz

**Affiliations:** 1 European Molecular Biology Laboratory, Cell Biology and Biophysics Unit, Meyerhofstraße 1, 69117 Heidelberg, Germany; 2 Max Planck Institute of Molecular Cell Biology and Genetics, Pfotenhauerstraße 108, 01307 Dresden, Germany; 3 Institute of Organic Chemistry and Biochemistry, Academy of Sciences of the Czech Republic, Flemingovo náměstí 2, 16610 Prague 6, Czech Republic; 4 Institut Interdisciplinaire de Neurosciences, CNRS UMR 5297 Université Bordeaux 2, 146, rue Léo-Saignat, 33077 Bordeaux, France

## Abstract

Photoactivation of caged biomolecules has become a powerful approach to study cellular signalling events. Here we report a method for anchoring and uncaging biomolecules exclusively at the outer leaflet of the plasma membrane by employing a photocleavable, sulfonated coumarin derivative. The novel caging group allows quantifying the reaction progress and efficiency of uncaging reactions in a live-cell microscopy setup, thereby greatly improving the control of uncaging experiments. We synthesized arachidonic acid derivatives bearing the new negatively charged or a neutral, membrane-permeant coumarin caging group to locally induce signalling either at the plasma membrane or on internal membranes in β-cells and brain slices derived from C57B1/6 mice. Uncaging at the plasma membrane triggers a strong enhancement of calcium oscillations in β-cells and a pronounced potentiation of synaptic transmission while uncaging inside cells blocks calcium oscillations in β-cells and causes a more transient effect on neuronal transmission, respectively. The precise subcellular site of arachidonic acid release is therefore crucial for signalling outcome in two independent systems.

## Abstract (web summary)

Caged signalling intermediates are powerful cell biological tools, however it can be challenging to precisely control where activation occurs. Nadler *et al*. develop a caging group that specifically targets the plasma membrane, and demonstrate spatially controlled activation of arachidonic acid signalling.

## Graphical abstract

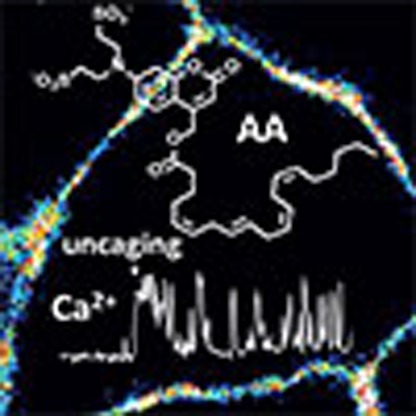

Cellular signalling networks are crucially dependent on small molecules. The molecular mechanisms involved in the respective signalling events are highly diverse and include direct interactions with target proteins, rapid concentration changes of the respective signalling molecules, intracellular concentration gradients and secondary signalling events because of ongoing metabolism^1^,^2^. Therefore, it is essential to mimic or modulate such events with high spatial and temporal precision. This is not always easily achieved by traditional approaches such as RNA interference or small-molecule inhibition of key enzymes^3^,^4^. Successful approaches for manipulating the cellular levels of small signalling molecules within a millisecond to second timeframe usually involve either chemical^5^,^6^ or optogenetic^7^,^8^ protein modulation systems for generating second messengers *in situ* or photoactivatable (caged) small molecules, which release the active species upon illumination^9^,^10^. Prominent examples of caged small molecules that have been employed in cell biology include lipids, nucleotides and neurotransmitters^11^,^12^,^13^,^14^,^15^,^16^,^17^. One major advantage of utilizing caged compounds is the possibility of stepwise concentration increases of signalling molecules to a fixed level from which they are subsequently metabolized. Only a few other methods enable these intracellular ‘relaxation' experiments, such as reversible dimerizer systems introduced by our group^18^ and others^19^,^20^,^21^ and switchable optogenetic protein modulation systems^22^. These approaches constitute valuable tools, but in most cases there are significant experimental challenges yet to overcome^10^. Uncaging assays for instance are still hindered by the difficulty of measuring the reaction progress of photoreactions at the single-cell level in the midst of live-cell imaging experiments and an almost complete lack of strategies to achieve organelle-specific compound release. To date, two-photon uncaging might be considered as one of the most promising approaches to confine an uncaging event to a distinct organelle^23^ and a number of novel caging groups with vastly improved optical properties have been developed over the last years^24^,^25^,^26^. Caged fluorophores offer probably the best approximation of an experimental tool to measure the progress of a photoreaction in living cells^27^. However, quantifying the photo-release of cellular messengers based on this approach is often difficult.

Stringent temporal and spatial control of externally induced signalling events is of particular importance if the investigated process is in part governed by highly dynamic concentration changes of small molecules. Arachidonic acid (AA) signalling provides a particularly striking example for the challenges of investigating small-molecule events. AA is directly involved in various cellular processes, most notably in controlled cell death (that is, through apoptosis), and by regulating vesicle fusion events occurring in neurite and axonal outgrowth, neurotransmitter release and insulin secretion^28^,^29^,^30^,^31^. Its direct molecular targets include a number of ion channels, syntaxin, protein kinase C and the G-protein-coupled receptor GPR40 (refs 32, 33, 34, 35, 36). Furthermore, AA may serve both as a second messenger in intracellular signalling cascades and as a messenger in intercellular communication^28^. Thus, the experimental possibility to alter AA concentrations at defined locations on a subcellular scale would be greatly beneficial for detailed understanding of its diverse cellular signalling roles. To address this, we developed a sulfonated caging group that allows for (i) uncaging of signalling lipids exclusively at the plasma membrane and (ii) straightforward quantification of uncaging reactions in living cells and the liberation of defined amounts of active compound. Here we demonstrate the applicability of the novel photoactivatable tool in two independent biological systems.

## Results

### Prelocation of caged signaling molecules at the plasma membrane

**Figure 1:**
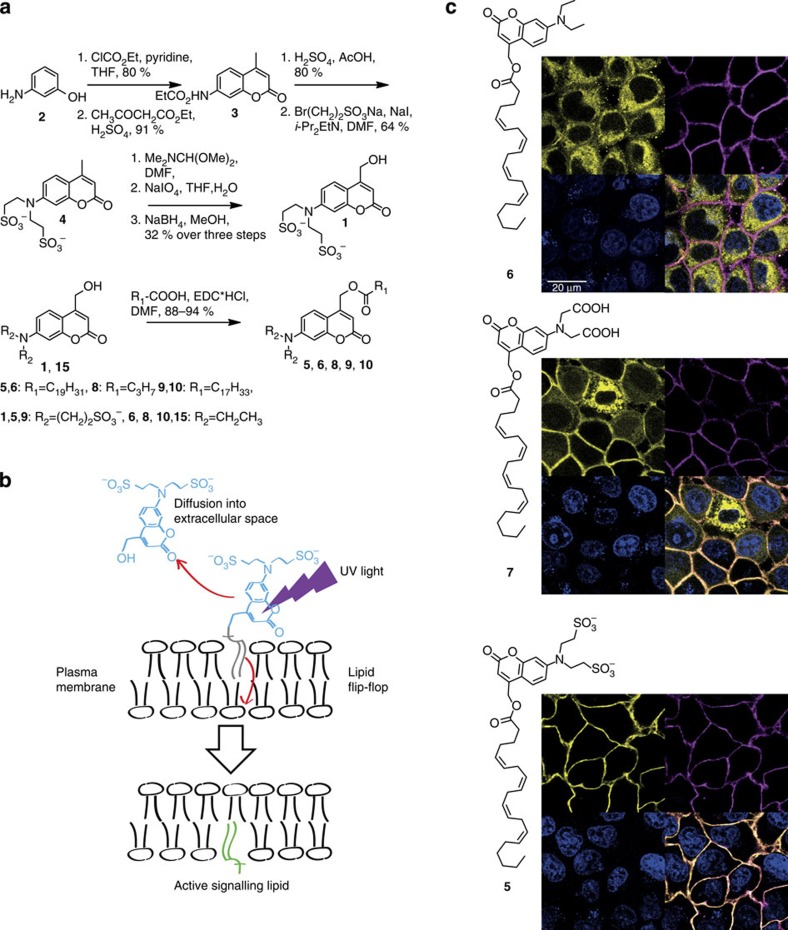
Synthesis and cellular localization of caged AA derivatives. (**a**) Synthesis of the sulfonated coumarin alcohol **1** and caged FAs. (**b**) Schematic representation of plasma membrane-specific photoactivation of caged signalling lipids. (**c**) Cellular localization of AA derivatives **6** (top), **7** (middle) and **5** (bottom) with different coumarin cages (coumarin—yellow, plasma membrane marker—magenta, nuclear marker—blue). Images were acquired using identical acquisition settings directly after removing loading solutions. All images are presented at the same magnification; the scale bar indicates 20 μm.

A set of caged fatty acid (FA) derivatives (**5**–**10**) was synthesized to study the consequences of rapidly elevated AA concentrations at the plasma membrane versus internal membranes (Supplementary Scheme 1 in the Supplementary Information). To achieve a stable pre-localization at the outer leaflet of the plasma membrane, we functionalized a photocleavable 7-diethylamino coumarin with two sulfonate groups (Fig. 1a). Their negative charges rendered the caged FA membrane-impermeant without significantly affecting the photophysical properties and the kinetics of the photoreaction (Supplementary Figs 1 and 2). Illumination removed the negative charges and enabled FA flip-flop across the plasma membrane, a fairly fast process for hydrophobic signalling lipids^37^ (Fig. 1b), and binding to interacting proteins. The sulfonated coumarin alcohol **1** was obtained in a reaction sequence starting from 3-aminophenol (**2**), which was converted into coumarin **3** in two steps. Cleavage of the carbamate protecting group and alkylation of the 7-amino function gave the sulfonated derivative **4**, which was subsequently oxidized to alcohol **1** (Fig. 1a). **1** was coupled to AA to yield the desired caged AA derivative **5**. We further synthesized the neutral variant **6** caged with the standard 7-diethylaminocoumarin caging group and a third compound **7** bearing two additional carboxylates instead of the sulfonate groups. The latter compound enabled us to establish that sulfonate groups are indeed crucial for efficient pre-localization at the plasma membrane (Fig. 1c). Finally, caged butyric acid (**8**) was synthesized as biologically inactive control compound and oleic acid (OA) derivatives **9** and **10** were prepared to distinguish AA signalling properties from other long-chain FAs (Fig. 1a and Supplementary Note).

The cellular localization of the respective caged AA derivatives **5**–**7** was analysed by confocal fluorescence microscopy in HeLa cells, taking advantage of the intrinsic fluorescence of all three coumarin caging groups. Compound **6** predominantly stained internal membranes in an unspecific manner, whereas the sulfonated derivative **5** was exclusively localized at the plasma membrane (Fig. 1c and Supplementary Information). Staining caused by compound **7** appeared to be fairly unstable and was difficult to reproduce (Fig. 1c and Supplementary Fig. 3b). Therefore, compound **7** was not used in cell experiments. The localization pattern of **5** was stable over a wide concentration range in the loading solution (Supplementary Fig. 4). We established that the photophysical properties of the relevant coumarin esters **5**, **6**, **7**, **8**, **9** and **10** were very similar (Supplementary Fig. 2) thus enabling us to use coumarin fluorescence to quantify lipid loading. However, the efficiency of membrane incorporation varied significantly for the different compounds. This prompted us to adjust the concentrations (15 μM for **5**, 100 μM for **6**) to be able to differentiate the physiological consequences of uncaging a given lipid at the plasma membrane or in internal membranes while excluding simple concentration effects (Supplementary Fig. 5a,b). The final cellular concentration of the caged AA derivatives was estimated to be 81±13 μM (**6**) and 67±17 μM (**5**), respectively (Supplementary Fig. 5e), well in line with reported AA levels in β-cells^28^,^38^,^39^. Next, we addressed the stability of the plasma membrane localization of the caged AA derivative **5**. We found that the amount of vesicular structures bearing the fluorophore was negligible in the first 30 min after exchanging the loading solution for the imaging medium and started to increase slowly afterwards (Supplementary Fig. 6), suggesting a time window of 30–60 min for generating consistent data.

### Quantification of the uncaging reaction in living cells

**Figure 2:**
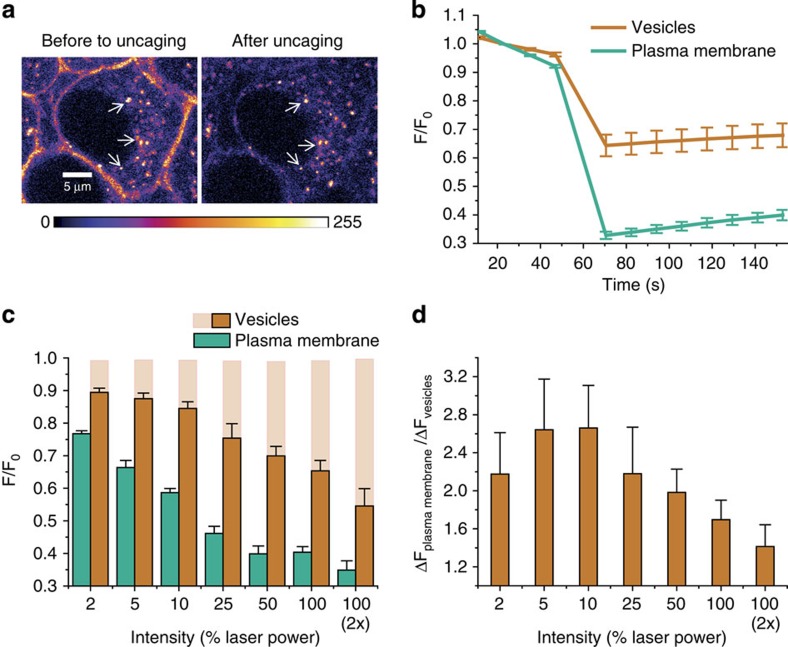
Quantifying photoreactions in living cells by uncaging of **5**. Cells were loaded with **5** and kept at 37 °C for 90–180 min after removal of the loading solution to ensure sufficient vesicle formation by endocytosis. (**a**) Distinctly different decreases of fluorescence intensity were observed at the plasma membrane and in vesicles upon photoactivation. The scale bar indicates 5 μm. (**b**) Quantification of normalized fluorescence intensity for the plasma membrane and vesicular structures over time for typical uncaging experiments (photoactivation settings: 1 scan, 50% 405 nm laser intensity, three individual experiments, error bars represent s.d.). (**c**) Remaining fluorescence intensity at the plasma membrane and in vesicular structures upon photoactivation with varied light intensity (error bars represent s.e.m., seven individual experiments). The shaded area represents the detected fluorescence intensity in vesicular structures before photoactivation; the observed difference is a measure for photobleaching. (**d**) Ratio of ΔF values determined for plasma membrane and vesicular fluorescence as a measure of photoreaction efficiency (errors derived by error propagation using the s.e.m. of the respective ΔF values, seven individual experiments).

We reasoned that a mixed localization of **5** at the plasma membrane and in vesicular structures might be very helpful for developing an assay for quantifying the efficiency of uncaging reactions in living cells. To generate such a mixed localization, we kept the cells at 37 °C for 90–180 min after removal of the loading solution to ensure sufficient vesicle formation by endocytosis. The predominant mechanism for the observed decreases in fluorescence intensity upon uncaging is inherently different for vesicles and the plasma membrane. Photoactivation of **5** at the outer leaflet of the plasma membrane releases the highly hydrophilic coumarin alcohol **1** into the extracellular space where it is readily dispersed by diffusion. The opposite holds true for the intra-vesicular localization. Diffusion of the photo-generated coumarin alcohol **1** is in this case limited by the size of the respective vesicular structure and its fluorescence will therefore continue to contribute to the observed signal. Lower fluorescence intensity after uncaging is in this case only caused by true photobleaching pathways. As expected, 90–180 min after loading to HeLa cells, we observed a pronounced reduction of fluorescence after uncaging **5** at the plasma membrane as compared with vesicular structures (Fig. 2a and Supplementary Movie M1). We developed a semi-automated image analysis approach to quantify this effect and thus established a general protocol for optimizing the required light intensity for uncaging experiments (Supplementary Methods and Supplementary Figs 7 and 8). The applicability of this protocol was exemplarily shown for two laser lines (375 and 405 nm) on a dual scanner confocal microscope (Olympus Fluoview 1200). Optimal laser settings were determined by systematic variation of the applied laser intensity (Fig. 2b,c and Supplementary Fig. 8). Although the normalized fluorescence intensity detected at the plasma membrane served as readout for judging the completeness of the photoreaction (Fig. 2c), the ratio of the observed decreases in fluorescence intensity at the plasma membrane and in vesicular structures was employed as a measure to determine laser settings for maximal efficiency of the photoreaction (Fig. 2d and Supplementary Fig. 8). In the example illustrated in Fig. 2, complete uncaging is reached between 25 and 50% laser intensity, whereas the highest efficiency is achieved between 5 and 10% laser intensity.

### Modulation of Ca^2+^ oscillations depends on the site of AA photorelease

**Figure 3:**
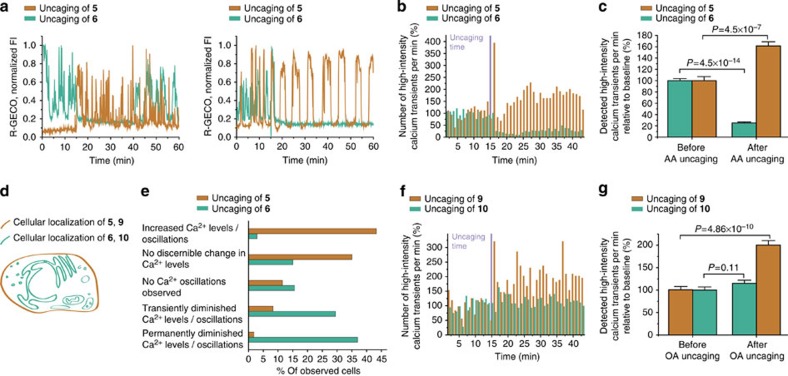
Compartment-dependent modulation of intracellular calcium dynamics in glucose-stimulated β-cells by fatty acids. (**a**) Exemplary calcium traces after uncaging compounds **5** and **6**, respectively; left: initiated and transiently interrupted Ca^2+^ oscillations; right: potentiated and terminated Ca^2+^ oscillations. (**b**) Number of detected high-intensity calcium events within every 60 s interval before and after uncaging compounds **5** and **6**. (**c**) Distribution of high-intensity calcium events (⩾60% of highest event in each trace) in ∼390 cells over time before and after AA uncaging from compounds **5** or **6**. Note: a detailed discussion of scope and limitations of the data analysis approach may be found in the Supplementary Fig. 10. *P*-values were obtained by Welch two sample *t*-test. (**d**) Schematic representation of cellular localization of compounds **5** and **6** before uncaging. (**e**) Classification of cellular responses after uncaging AA. (**f**) Number of detected high-intensity calcium events within every 60 s interval before and after uncaging oleic acid (OA) from compounds **9** and **10**. (**g**) Distribution of high-intensity calcium events (⩾60% of highest event in each trace) in 304 cells before and after uncaging **9** and **10**. Error bars represent s.e.m. throughout the figure.

We chose to study two processes known to involve AA signalling, namely lipid-induced potentiation of hippocampal mossy fibre (Mf) synaptic transmission and insulin secretion by pancreatic β-cells in order to demonstrate the potential of the method for establishing distinct roles of signalling lipids in different cellular compartments. AA was shown to be present in fairly high concentrations (50–75 μM) in pancreatic β-cells, to modulate calcium release, and as a result to trigger insulin secretion in β-cells^28^,^38^,^39^. Similarly, artificially reduced levels of AA led to impaired insulin secretion^40^. However, a conclusive model describing the interplay between AA concentration and insulin secretion has not been proposed so far and our understanding of its molecular targets and their intracellular localization remains incomplete. We chose the mouse insulinoma-derived cell line MIN6 as a model β-cell line to perform live-cell uncaging experiments. These cells exhibit characteristics of glucose metabolism and glucose-stimulated insulin secretion similar to those observed for islets^41^. When exposing MIN6 cells expressing the genetically encoded Ca^2+^ sensor R-GECO^42^ to a glucose concentration of 20 mM, we observed characteristic calcium oscillations, which subsequently triggered increased insulin release (Supplementary Fig. 9). Upon addition of free AA, the average calcium concentration decreased markedly and calcium oscillations were significantly diminished (Supplementary Fig. 10a,b). On the contrary, the addition of compounds **5** and **6** did not have significant long-term effects on calcium oscillations, thereby establishing that both caging groups sufficiently mask the AA moiety to block its cellular signalling activity (Supplementary Fig. 10c,d). As observed for HeLa cells, incubation of MIN6 cells with **5** resulted in a pronounced plasma membrane stain, whereas **6** stained internal membranes in a homogenous manner (Supplementary Fig. 11). To study the effects of elevated AA levels at internal membranes versus the plasma membrane, we performed AA uncaging experiments under optimized conditions (Supplementary Fig. 8) with compounds **5** and **6** in 390 cells while monitoring changes in the intracellular Ca^2+^ concentration using the Ca^2+^ sensor R-GECO. As the analysed data set comprised a large cell population exhibiting widely diverse calcium oscillation patterns with regard to frequency, amplitude and duration of calcium events, we analysed the obtained data set by several independent approaches addressing the distribution of individual calcium events over the time course of all experiments. To this end, we employed a peak detection algorithm (‘Package Peaks; R' see Supplementary Information for details) and identified 6,808 individual events, which were included in the analysis. By conducting an internal normalization, we used the relative height of each event with regard to the highest detected peak in each trace as a criterion to group all calcium transients into high- (intensity ⩾60% of highest peak) and low-intensity (intensity <60% of highest peak) events. Uncaging of compounds **5** and **6** resulted in surprisingly different changes in the observed distribution of high-intensity calcium events (Supplementary Movies M2 and M3). Uncaging AA from **5** at the plasma membrane triggered an immediate fourfold increase in high-intensity calcium events. This acute effect was not observed after uncaging AA from **6** (Fig. 3b and Supplementary Fig. 12). In addition, high-intensity calcium events were detected with a permanently augmented rate after uncaging AA from **5**, whereas uncaging AA from **6** had the opposite effect, that is, blocked or reduced Ca^2+^ oscillations (Fig. 3b,c, Supplementary Fig. 10 in the Supplementary Information). These findings imply that solely higher levels of AA at the plasma membrane as opposed to elevated concentrations at internal membranes are responsible for increased calcium signalling in β-cells.

In an effort to gain further insight into the nature of the observed effects, we classified the cells according to their response pattern and compared the distribution of cellular responses after liberating AA at the plasma membrane or internal membranes. This classification was performed both by manual assignment (blind expert assignment) and an automated approach based on a newly developed algorithm for analysing calcium oscillations (Supplementary Methods) with both methods yielding very similar results (Fig. 3e and Supplementary Fig. 13). After liberating AA at the plasma membrane by uncaging **5**, we observed prolonged increases of the average calcium concentration as well as permanently enhanced amplitudes in a significant fraction of the analysed cells. The duration of individual calcium events was often increased. Furthermore, calcium oscillations were initiated in a number of cells that did not exhibit an oscillatory behaviour before the uncaging event (Fig. 3a,e). On the contrary, AA release at internal membranes caused by uncaging of **6** typically resulted in transiently (typically 4–10 min) or permanently diminished calcium oscillations and lower average calcium levels after occasional initial calcium transients (Fig. 3a,e). These findings indicate a dual role for AA signalling in β-cells. Although enhanced levels of AA at the plasma membrane trigger and potentiate calcium oscillations and thereby ultimately cause enhanced insulin release, opposing effects are caused by higher AA levels at internal membranes.

We performed a series of control experiments to rule out potential side effects caused by the respective photoreactions or the released coumarin alcohol species. We first established that neither addition nor photoactivation of the sulfonated coumarin alcohol **1** or photoactivation of 7-diethylamino caged butyrate (**8**) as a biologically inactive control compound changed the observed pattern of Ca^2+^ oscillations in MIN6 cells (Supplementary Fig. 14a–c). It can thus be concluded that the observed effects were neither artefacts caused by the photoreactions nor due to release of coumarin alcohol or its further metabolites.

To assess whether the observed dual signalling role is a general response of MIN6 cells to elevated levels of unsaturated FAs at the plasma membrane versus internal membranes, we prepared caged OA derivatives bearing the novel sulfonated (**9**) or the neutral cage (**10**; Fig. 1a) and uncaged them in MIN6 cells. We found that the modulation of Ca^2+^ oscillations triggered by uncaging of OA at the plasma membrane was similar to the effect observed for AA uncaging, namely an initial burst of high-intensity events directly after uncaging followed by long-lasting enhanced calcium signalling (Fig. 3f,g). Importantly, uncaging of OA at internal membranes did not have any noticeable effect on calcium oscillations (Fig. 3f,g).

**Figure 4:**
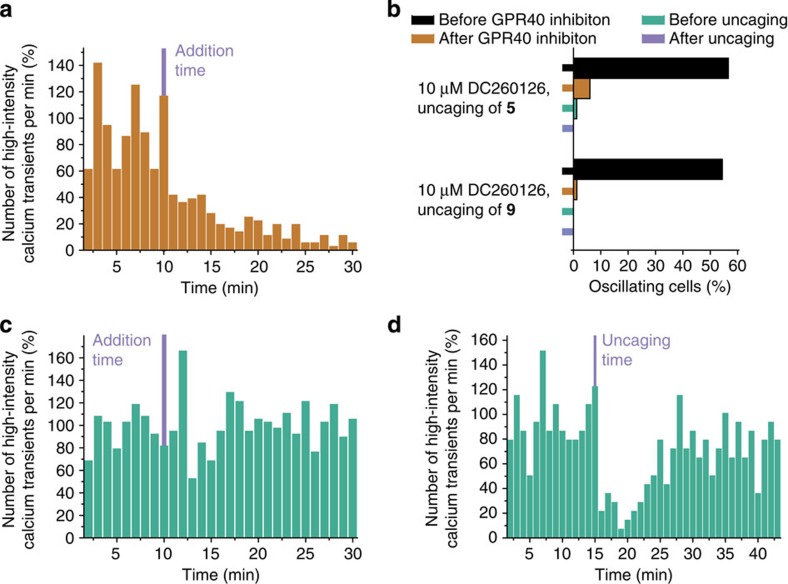
Effects of GPR40 and CB1 inhibition on intracellular calcium dynamics in glucose-stimulated β-cells. (**a**) Number of detected high-intensity calcium events within every 60 s interval before and after addition of the specific GPR40 inhibitor DC260126 (10 μM) in 162 cells. (**b**) Percentage of oscillating cells before and after DC260126 addition as well as before and after subsequent uncaging of OA and AA at the plasma membrane using compounds **5** and **9**, respectively. Two time lapses were recorded in the same field of view with a 15-min delay to ensure a stable baseline after inhibitor treatment and before lipid uncaging. (**c**) Number of detected high-intensity calcium events within every 60 s interval before and after addition of the specific CB1 inhibitor AM251 (100 nM) in 141 cells. (**d**) Number of detected high-intensity calcium events within every interval of 60 s before and after uncaging AA at internal membranes (**6**) in cells pre-treated with 100 nM AM251 in 63 cells.

The effects of higher AA and OA levels on the plasma membrane are well in line with previous studies^35^,^38^,^39^ and are probably caused by direct interaction of AA and other FAs with a number of signalling effectors, most likely the pancreatic islet GPR40 (refs 32, 33, 34, 35, 36), which has been shown to be indiscriminately activated by different FAs^35^. After confirming GPR40 expression in MIN6 cells (Supplementary Fig. 15), we evaluated whether inhibition of GPR40 influenced Ca^2+^ oscillations or the modulation of oscillation patterns caused by AA and OA uncaging at the plasma membrane. When applying the GPR40 inhibitors DC260126 (refs 43, 44; 10 μM) or GM1100 (ref. 45; 10 μM), we found a gradual disappearance of Ca^2+^ oscillations (Fig. 4a and Supplementary Fig. 14d). We next performed uncaging experiments of **5** or **9** in MIN6 cells after treatment with 10 μM DC260126. Manual classification of Ca^2+^ traces by blind expert assignment revealed that AA or OA uncaging failed to restore Ca^2+^ oscillations to pre-inhibition levels (Fig. 4b) or even augment oscillatory behaviour relative to the lower post-inhibition baseline. Taken together, these data confirm that GPR40 serves as one of the main plasma membrane receptors for FA-induced Ca^2+^ signalling. In fact, it appears that a basal activation of GPR40 by the autocrine release of FAs is required for maintaining Ca^2+^ oscillations in β-cells as addition of lipid-free bovine serum albumin led to diminished oscillatory behaviour (Supplementary Fig. 14e).

Alternatively, metabolites such as endocannabinoids could be essential players in driving calcium oscillations. We therefore inhibited the CB1 receptor by adding the specific inhibitor AM251 (100 nM), which did not alter oscillation patterns in any discernible way (Fig. 4c). Uncaging AA (**6**) on internal membranes of MIN6 cell in the presence of 100 nM AM251 (refs 46, 47) also led to a transient reduction of Ca^2+^ oscillations (Fig. 4d), indicating that acute reduction of high-intensity calcium events is probably not due to endocannabinoid signalling but rather a direct effect of higher AA concentrations. These findings indicate that elevated levels of unsaturated FAs at the plasma membrane constitute a general cue for enhanced, likely GPR40-mediated Ca^2+^ signalling activity. When uncaged at internal membranes, elevated levels of unsaturated FAs lead to different effects depending on the FA structure. AA caused downregulation of Ca^2+^ oscillations, whereas another long-chain unsaturated FA (OA) had no significant effect on Ca^2+^ oscillations.

### AA release at the plasma membrane potentiates synaptic transmission

**Figure 5:**
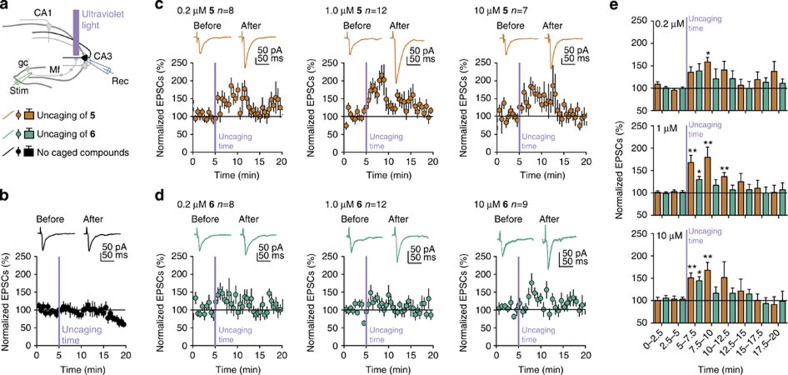
Potentiation of mossy fibre (Mf) synaptic transmission following photorelease of AA at the plasma membrane versus internal membranes. (**a**) Schematic representation of a hippocampal slice with recording (blue, in a CA3 pyramidal cell) and stimulating electrodes (green, inside the dentate gyrus). Slices were perfused with compound **5** or **6** for 10–15 min before flashing ultraviolet light in the *stratum lucidum* near the recorded CA3 pyramidal cell. gc, granule cell; rec, recording (voltage clamp recording of excitatory post synaptic currents); stim, stimulation (stimulation of the granule cell axons). (**b**) Control experiment performed in the absence of caged compounds excluded photoartefacts on synaptic transmission. Upper panel: sample traces (average of 30 sweeps) of mossy fibre (Mf) EPSCs before and after uncaging. Lower panels: summary time course of normalized Mf-EPSCs. (**c**) Modulation of Mf-EPSCs after uncaging compound **5** (0.2, 1.0 or 10 μM). Upper panels: sample traces (average of 30 sweeps) of Mf-EPSCs before and after uncaging. Lower panels: summary time course of normalized Mf-EPSCs. (**d**) Modulation of Mf-EPSCs after uncaging compound **6** (0.2; 1.0 or 10 μM). Upper panels: sample traces (average of 30 sweeps) of Mf-EPSCs before and after uncaging. Lower panels: summary time course of normalized Mf-EPSCs. (**e**) Bar graphs illustrating the different time regimes of the effects illustrated in **c**,**d**. Error bars represent s.e.m. *P*-values were obtained by Wilcoxon matched pairs test. *P<0.05, **P<0.01.

In an attempt to establish whether different roles for AA signalling at individual organelles are a more widespread phenomenon, we compared the efficiency of AA release from compounds **5** and **6** with regard to synaptic transmission in hippocampal brain slices prepared from C57B1/6 mice. Utilizing this experimental model allowed us to demonstrate the applicability of caged signalling lipids equipped with the novel sulfonated caging group in relatively thick (300 μm) tissues. We chose to study mossy fibre to CA3 synapses where the strength of synaptic transmission is tightly controlled by presynaptic Kv channels^48^,^49^, a known sensitive target of AA^32^. We recently established that AA acts as a retrograde messenger at these synapses by broadening presynaptic action potentials mainly due to direct inactivation of presynaptic Kv channels at the plasma membrane^30^. In the current paper, we used compounds **5** and **6** to study the dynamic of this effect in greater detail. We performed whole-cell voltage clamp recordings of excitatory post synaptic currents (EPSCs) from visualized CA3 pyramidal cells by stimulating the granule cell axons (mossy fibres) to release glutamate from synaptic vesicles (Fig. 5).

As previously observed for **6** (ref. 30), uncaging of AA from **5** and **6** at 10 μM loading concentration induced robust and transient potentiation of synaptic transmission. Loading of both compounds was confirmed by fluorescence microscopy (Supplementary Fig. 16). Application of the uncaging protocol without compound loading had no effect on synaptic transmission, indicating that illumination alone does not alter major electrophysiological properties (Fig. 5b). Importantly, the duration of this effect was markedly dependent on the specific nature of the membranes where AA photorelease took place. Although release of AA at the plasma membrane resulted in prolonged potentiation of synaptic transmission irrespective of the applied concentration (7.5–10 min, Fig. 5e), release at internal membranes only sparked a brief potentiation, which was potentially caused by diffusion of a minute amount of released AA towards its plasma membrane target (Fig. 5c–e). This is in line with the proposed mechanism for the action of AA as a retrograde messenger via Kv channels, located at the plasma membrane.

## Discussion

The number of approaches for organelle-specific localization of photocaged small signalling molecules is extremely limited^50^,^51^ and no method for directly targeting the plasma membrane has been reported so far. We have developed a novel caging group that allows photorelease of hydrophobic signalling molecules exclusively at the outer leaflet of the plasma membrane. To the best of our knowledge, this is the first example of organelle-specific photorelease of cellular signalling lipids achieved by controlling the cellular localization of the caged compound before photoactivation. In addition, this sulfonated caging group is a suitable tool for quantifying the completeness and efficiency of photoreactions inside living cells, thereby greatly facilitating the setup and optimization of uncaging experiments and simultaneously reducing the need for a functional readout. We applied the synthesized caged AA derivatives to study AA signalling with subcellular precision both in cell culture and tissue. Our results suggest that the initial cellular site of increased AA concentration plays a crucial role in determining signalling outcome in very different cellular settings despite the fact that AA is a readily diffusible small molecule. Our findings underline the intricate nature of the molecular mechanisms governing small-molecule signalling and also the growing need to develop more sophisticated tools for its study. Caged small molecules that can be released in an organelle-specific manner will likely play an important role both as discovery tools and by providing a straightforward approach to analysing metabolism and lifetimes of small-molecule messengers at distinct subcellular sites.

## Methods

### General synthetic procedures

All chemicals were obtained from commercial sources (Acros, Sigma, Aldrich, Enzo, Lancaster or Merck) and were used without further purification. Solvents for flash chromatography were obtained from VWR and dry solvents were obtained from Sigma. Deuterated solvents were obtained from Deutero GmbH. All reactions were carried out using dry solvents under an inert atmosphere unless otherwise stated in the respective experimental procedure. Thin-layer chromatography was performed on precoated plates of silica gel (Merck, 60 F254) using ultraviolet light (254 or 366 nm) or a solution of phosphomolybdic acid in EtOH (10 g phosphomolybdic acid, in 100 ml EtOH) for analysis. Preparative column chromatography was performed using silica from Merck (silica 60, grain size 0.063–0.200 mm) with a pressure of 1–1.5 bar. HPLC was performed on a Knauer HPLC Smartline Pump 1000 using a Knauer Smartline UV Detector 2,500 instrument. A 250mm × 10 mm LiChrospher 100 RP-18 column was used for semi-preparative HPLC applications. ^1^H- and ^13^C-NMR-spectra were obtained on a 400-MHz Bruker UltraShield spectrometer. Chemical shifts of ^1^H- and ^13^C-NMR-spectra are referenced indirectly to tetramethylsilane. *J* values are given in Hz and chemical shifts in p.p.m. Splitting patterns are designated as follows: s, singlet; d, doublet; t, triplet; q, quartet; m, multiple; b, broad. ^13^C-NMR-spectra were broadband hydrogen decoupled. Mass spectra (ESI) were recorded using a Waters Micromass ZQ mass spectrometer or a HP Esquire-LC mass spectrometer. High-resolution mass spectra (HRMS) were recorded at the University of Heidelberg.

### Brief description of conducted chemical syntheses

*N*-Ethylcarbamoyl-3-aminophenol **11** was condensed with ethyl acetoacetate in the presence of sulfuric acid to afford coumarin precursor **3**, which after deprotection with 1:1 sulfuric acid/glacial acetic acid mixture was alkylated with sodium 2-bromoethanesulfonate in *N,N*-dimethylformamide. The bis-sulfonated, water-soluble 7-amino-4-methylcoumarin derivative **4** was purified by reverse phase chromatography employing a triethylammonium bicarbonate buffer system and obtained as its bis-triethylammonium salt. The 4-methyl group was converted to the alcohol using a previously reported protocol^52^ in three steps. Esters were prepared from the alcohol in a carbodiimide-mediated reaction utilizing AA or OA to give compounds **5** and **9**, respectively. The target compounds were obtained as readily soluble triethylammonium salts from reverse phase chromatography. Additional neutral or carboxylated coumarin precursors were synthesized according to the literature protocols^53^,^54^. They were coupled to the respective carboxylic acids using carbodiimide-mediated reactions, which yielded coumarin esters **6**, **8**, **10** and **17**. The synthesis of **6** was published previously by our group^30^. The *t*-Bu protecting groups of **17** were removed by treatment with trifluoroacetic acid. A detailed description of the conducted syntheses as well as NMR and MS data of all new compounds may be found in the Supplementary Information.

### Photophysical characterization of new compounds

Absorbance spectra were measured using a Cary 60 UV–vis spectrophotometer (Agilent Technologies) in Cary WinUV Scan Application (version 5.0.0.999). The detection range was set to 250–500 nm, the spectral resolution to 0.5 nm and the averaging time to 0.1 s. The path length of the cuvette was 1 cm, baseline correction was carried out by subtraction of the background signal of an EtOH sample and the compound absorbance maxima were below 0.1.

All emission spectra were measured using a Photon Technology International Fluorimeter in FeliX32 Analysis Application (version 1.2). The excitation wavelength was set to 360 nm and emission collection to 370–700 nm with a step size of 3 nm and 0.1 s integration. Spectra were recorded from EtOH solutions and averaged three times. Emission end excitation slits were set to 2 nm (0.5 mm W). Quantum yields (QYs) were calculated as following:


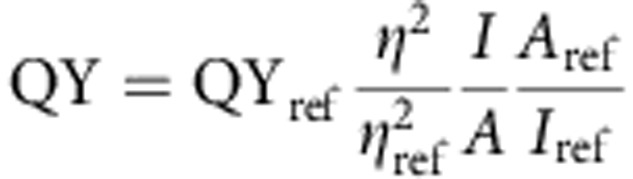


where QY_ref_ is the QY of the reference compound (coumarin 1, QY=0.73 (ref. 55)), *η* is the refractive index of the solvent (ethanol for all samples and the reference), *A* is the absorbance at the excitation wavelength, *I* is the integrated fluorescence intensity.

### Cell culture and cDNA transfection

MIN6 cells were kindly provided by the Miyazaki laboratory. HeLa ‘Kyoto' cells were obtained from the Kyoto University. The initial characterization of caged lipid performance in cells, loading and localization analyses as well as development and optimization of uncaging protocols and quantification of photoreactions were carried out in HeLa ‘Kyoto' cells. Cells were grown in low-glucose DMEM (31885-023, Life Technologies) supplied with 10% fetal bovine serum (F7524, Sigma) and 100 μg ml^−1^ antibiotic Primocin (ant-pm-1, Invitrogen). Cells were seeded in eight-well Lab-Tek microscope dishes 24–48 h (to reach 50–80% confluence) before imaging. The mouse insulinoma-derived cell line MIN6 used in this study as a model β-cell line was initially developed and kindly provided by Miyazaki *et al*.^56^. Cells were grown in high-glucose DMEM (41965-039, Life Technologies) supplied with 15% fetal bovine serum (10270098, Lifetechnologies), 100 U ml^−1^ of antibiotics penicillin-streptomycin (15140122, Life Technologies) and 70 μM β-mercaptoethanol, always added freshly to the cell culture flasks (P07-05100, PAN-Biotech). Cells were seeded in eight-well Lab-Tek microscope dishes 48–64 h (to reach 50–80% confluence) before imaging. For calcium imaging, MIN6 cells were transfected with cDNA coding for the R-GECO Ca^2+^ reporter and cDNA coding for the C1-GFP DAG sensor (as a control) usually 24–48 h after seeding. A transfection cocktail of 200 ng C1-GFP and 200 ng R-GECO in 10 μl of Opti-MEM (31985-070, Life Technologies) and 1.5 μl of Lipofectamine 2000 transfection reagent (11668030, Life Technologies) was added to each well of an eight-well Lab-Tek microscope dish loaded with 200 μl Opti-MEM (37 °C, immediately before the addition) 24–48 h prior imaging.

### Fluorescence microscopy

Cells were imaged in eight-well Lab-Tek microscope dishes (155411, Thermo Scientific) at 37 °C in imaging buffer containing (mM): 20 HEPES, 115 NaCl, 1.2 CaCl_2_, 1.2 MgCl_2_, 1.2 K_2_HPO_4_. For HeLa cells, the imaging buffer additionally contained 10 mM glucose. MIN6 cells (if not specified otherwise) were imaged in an imaging buffer containing a stimulatory amount of glucose (20 mM).

Imaging was performed on a dual scanner confocal microscope Olympus Fluoview 1200, with × 20 (air) and/or × 63 (oil) objectives. This microscope houses two independent, fully synchronized laser scanners for simultaneous laser stimulation and confocal observation and permits capturing of cellular responses that occur during or immediately following laser stimulation. Microscope settings were adjusted to generate images displaying background fluorescence values slightly larger than zero in order to capture the complete signal stemming from the respective fluorescent dyes or proteins. Coumarin dyes were excited with 405 nm laser and emitted light was collected between 425 and 525 nm. C1-GFP and R-GECO were excited with 488 and 559 nm lasers and emitted light was collected at 500–550 nm and 570–670 nm, respectively.

### Image analysis and data processing

Images were analysed using Fiji ( http://fiji.sc/Fiji) and the previously reported FluoQ macro^57^ (for calcium imaging data) or the newly developed ImageJ macro ‘PM/background ratio-calculator Macro' (see Supplementary Methods for description of ImageJ macros and the general pipeline used for analysis of coumarin imaging data). Raw images were loaded into Fiji and the respective macro was started to perform all image analysis steps. An R script based on the package ‘peaks'^47^ was used to automatically detect calcium transients in obtained single-cell traces. A detailed description of the performed image analysis steps, a discussion of the chosen parameters and the source code of all newly developed Fiji macros and R scripts may be found in the Supplementary Information.

### Electrophysiology

Parasagittal hippocampal slices (320 μm thick) were obtained from 30- to 35-day-old C57Bl/6 mice. Slices were transferred to a recording chamber in which they were continuously superfused with an oxygenated extracellular medium (95% O_2_ and 5% CO_2_) containing (mM): 125 NaCl, 2.5 KCl, 2.3 CaCl_2_, 1.3 MgCl_2_, 1.25 NaH_2_PO_4_, 26 NaHCO_3_, 20 glucose, pH 7.4. Whole-cell recordings were made at ∼25 °C from CA3 pyramidal cells under infrared differential interference contrast imaging using borosilicate glass capillaries, which had resistances between 4 and 8 MΩ. For voltage-clamp recordings from CA3 pyramidal cells, the patch electrodes were filled with a solution containing (mM): 140 CsCH_3_SO_3_, 2 MgCl_2_, 4 NaCl, 5 phospho-creatine, 2 Na_2_ATP, 0.2 EGTA, 10 HEPES, 0.33 GTP, pH 7.3 adjusted with CsOH. Bicuculline (10 μM) was present in the superfusate of all experiments. A patch pipette (open tip resistance ∼5 MΩ (about 1 μm tip diameter)) was placed in the dentate gyrus to stimulate Mfs. Mf synaptic currents were identified according to the following criteria: robust low-frequency facilitation, low-release probability at 0.1 Hz, rapid rise times of individual EPSCs (∼1 ms) and EPSC decays free of secondary peaks that may indicate the presence of polysynaptic contamination. Fresh aliquots of caged AA derivatives **5** and **6** were used for each experiment and were dissolved in extracellular medium. The slices were perfused with extracellular medium containing either **5** or **6** for at least 10–15 min before starting the experiments to ensure homogenous penetration of the caged compound into the slice (Supplementary Fig. 16). During the application of caged AA derivatives **5** and **6**, a total amount of 10–15 ml of extracellular solution containing the caged compound was continuously re-circulated and oxygenated. AA was locally uncaged in the *stratum lucidum* of the patched CA3 pyramidal cell or the patched MfB by an ultraviolet flash photolysis (Xenon flash lamp; Rap OptoElectronic).

Statistical analysis was carried out as follows: Values are presented as mean±s.e.m. of *n* experiments. Nonparametric tests were used for statistical analysis. Within cell comparisons were made using the Wilcoxon matched pairs test, which was applied to raw non-normalized data of baseline values and values obtained after applying the respective uncaging protocol. The Mann–Whitney test was used for comparison of two groups. Statistical differences were considered as significant at *P*<0.05. Statistical analysis was performed using GraphPad prism software.

All animals were used according to the guidelines of the University of Bordeaux/CNRS Animal Care and Use Committee.

## Additional information

**How to cite this article:** Nadler, A. *et al*. Exclusive photorelease of signalling lipids at the plasma membrane. *Nat. Commun.* 6:10056 doi: 10.1038/ncomms10056 (2015).

## Supplementary Material

Supplementary Information Supplementary Figures 1-16, Supplementary Note 1 and Supplementary Methods

Supplementary Movie 1 Coumarin release after uncaging

Supplementary Movie 2 Calcium oscillations after uncaging arachidonic acid at the plasma membrane

Supplementary Movie 3 Calcium oscillations after uncaging arachidonic acid at internal membranes
