# Supplementary material for: Exclusive photorelease of signalling lipids at the plasma membrane
Source: Nat Commun. 2015 Dec 21;6:10056. doi: 10.1038/ncomms10056 (PMC4703838; doi:10.1038/ncomms10056)

# Supplementary figures

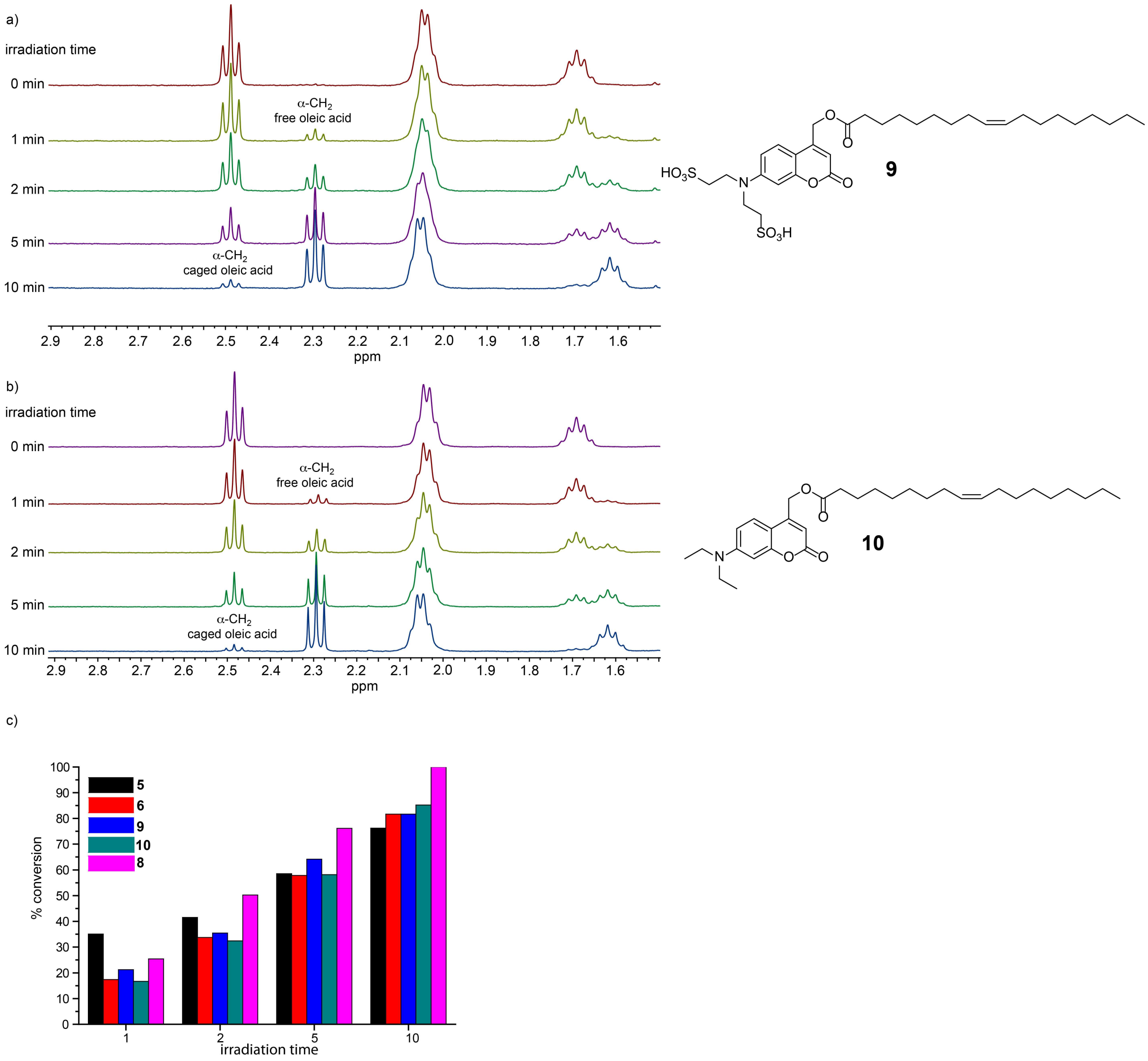


*Supplementary Figure 1. Comparison of photocleavage efficiency for compounds **5**, **6**, **8**, **9** and **10**.* Irradiation was carried out in MeOD-d4 using a Newport 66924 light source equipped with a 1000 W mercury arc lamp and a 350 nm highpass filter for defined amounts of time directly in the NMR tube. The photoconversion was monitored by the distinct shift of the fatty acid α-$CH_2$ group located at 2.5 (caged fatty acid) and 2.3 ppm (free fatty acid). a) Irradiation of compound **9** featuring the sulfonated cage. Left panel: aliphatic region of the NMR spectrum of the reaction mixture at defined time points. Right panel: Structure of compound **9**. b) Irradiation of compound **10** featuring the neutral cage. Left panel: aliphatic region of the NMR spectrum of the reaction mixture at defined time points. Right panel: Structure of compound **10**. c) Quantification of the uncaging efficiency for all compounds used in biological assays (**5**, **6**, **8**, **9**, **10**). Conversion rates were determined by integrating α-$CH_2$ signals of free and caged fatty acids at 2.3 and 2.5 ppm, respectively.

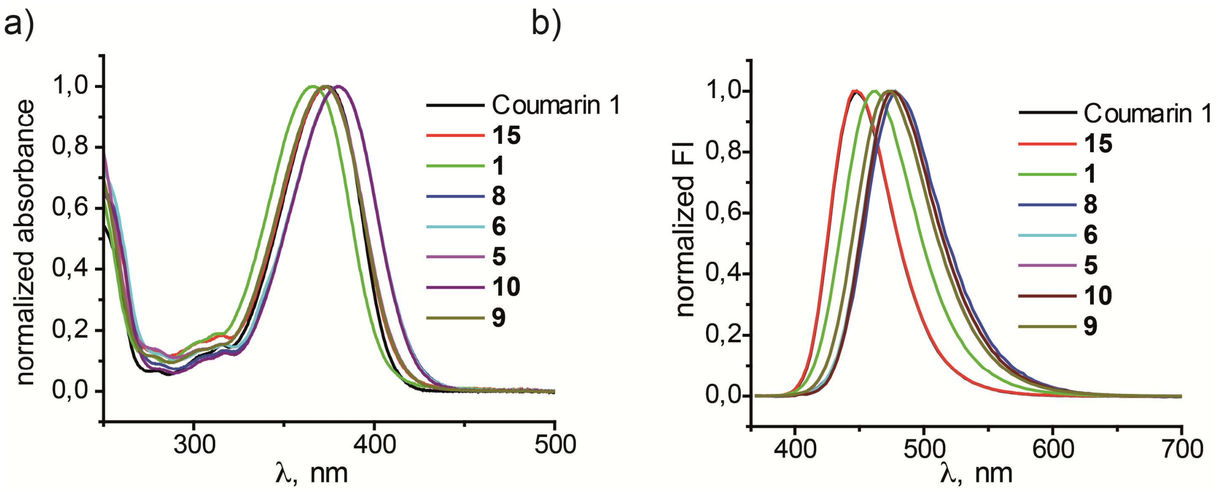


c)

| Compound | Absorption maximum (nm) | Extinction coefficient (L*mol$^{-1}$*cm$^{-1}$) | Quantum yield |
|---|---|---|---|
| Coumarin 1 | 376 | 23437 | 0.73 |
| **15** | 374 | 18826 | 0.58 |
| **1** | 366 | 19402 | 0.71 |
| **5** | 373 | 23166 | 0.48 |
| **6** | 381 | 21580 | 0.34 |
| **9** | 381 | 20880 | 0.50 |
| **10** | 373 | 16062 | 0.37 |
| **8** | 381 | 18396 | 0.19 |

*Supplementary Figure 2. Photophysical properties of coumarin derivatives* ***1****,* ***5****,* ***6****,* ***8****,* ***9****,* ***10*** *and* ***15****.* a) Absorption spectra of compounds **1**, **5**, **6**, **8**, **9**, **10** and **15**. b) Emission spectra of compounds **1**, **5**, **6**, **8**, **9**, **10** and **15**. c) Absorption maxima, extinction coefficients and fluorescence quantum yields of all relevant coumarin derivatives. Absorbance was measured using a Cary 60 UV-Vis spectrophotometer (Agilent Technologies) in Cary WinUV Scan Application (version 5.0.0.999) The detection range was set to 250-500 nm, the spectral resolution to 0.5 nm and the averaging time to 0.1 sec. The path length of the cuvette was 1 cm, baseline correction was carried out by subtraction of the background signal of an ethanol sample and the compound absorbance maxima were below 0.1. Extinction coefficients are given for the respective absorption maximum.
All emission spectra were measured using a Photon Technology International (PTI) Fluorimeter in FeliX32 Analysis Application (version 1.2). The excitation wavelength was set to 360 nm and emission collection to 370-700 nm, with a step size of 3 nm, and 0.1 s integration, Spectra were recorded from EtOH solutions, averaged 3 times, emission end excitation slits were set to 2 nm (0.5 mm W)

Quantum Yields (QYs) where calculated as following:

$$QY = QY_{ref}\frac{\eta^2}{\eta_{ref}^2}\frac{I}{A}\frac{A_{ref}}{I_{ref}} \quad \text{(I)}$$

where $QY_{ref}$ is the quantum yield of the reference compound (Coumarin 1, QY = 0.73, Jones G. *et al.*, *J. Phys. Chem.*, 294 (1985)), $\eta$ is the refractive index of the solvent (ethanol for all samples and the reference), $A$ is the absorbance at the excitation wavelength, and I is the integrated fluorescence intensity.

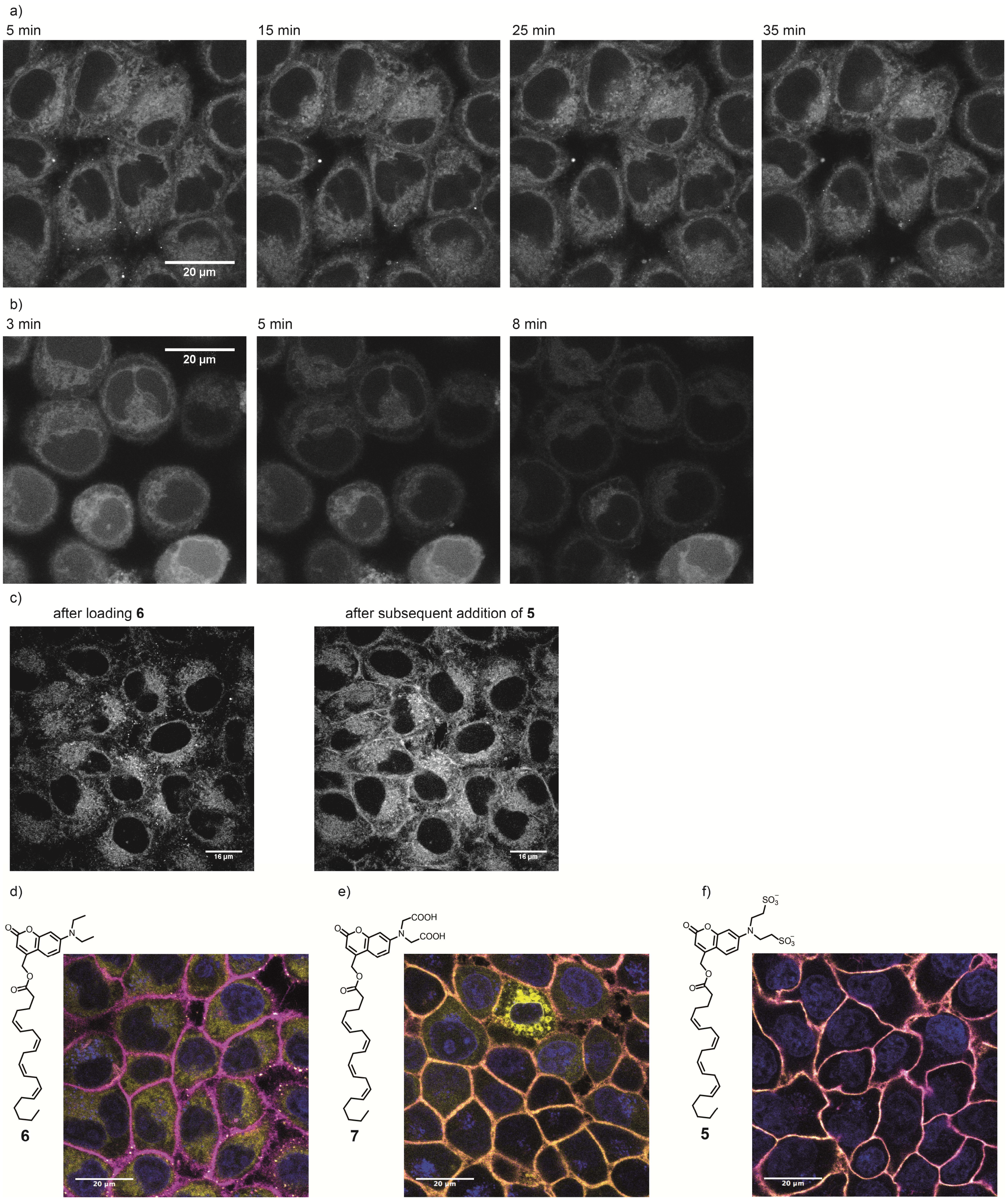


*Supplementary Figure 3. Cellular localization of caged compounds* a) Stability of internal membrane stain induced by compound **6**. HeLa cells were pre-incubated with 100 µM **6** at 25 °C for 20 min, the loading solution was subsequently removed and imaging buffer added. Images were acquired at 37 °C every 10 min using identical acquisition parameters. The detected fluorescence intensity and the intracellular localization remained stable throughout the imaging period. b) Stability of cellular stain induced by compound **7**. All parameters were the same as for a). Images were acquired at the indicated timepoints. The detected fluorescence intensity decreased markedly after removal of the loading solution and this process appeared to occur at different speed for individual cells. c) Subsequent

loading of **6** and **5** in the same cells using the loading conditions detailed above. Application of **5** to HeLa cells loaded with **6** results in the appearance of an additional plasma membrane stain of similar intensity compared to the previously observed internal membrane stain. d –f) 3D reconstructions of the confocal Z-stack images (taken every 0.37 micron) of HeLa cells loaded correspondently with compounds **6, 7** and **5** (in yellow) and stained with Plasma membrane marker (in magenta; CellMask, Lifetechnologies #C37608) and Nuclear marker (in blue; DRAQ5, Lifetechnologies #62251).The displayed cells are representative and the observed localization pattern did not vary across experiments for compounds **6** and **5**. In case of compound **7** the cytosolic fraction of the compound appeared to vary. The intracellular localization of **7** and the stability of the induced stain of cellular proved to be unpredictable. Therefore we decided not to apply this compound in biological assays.

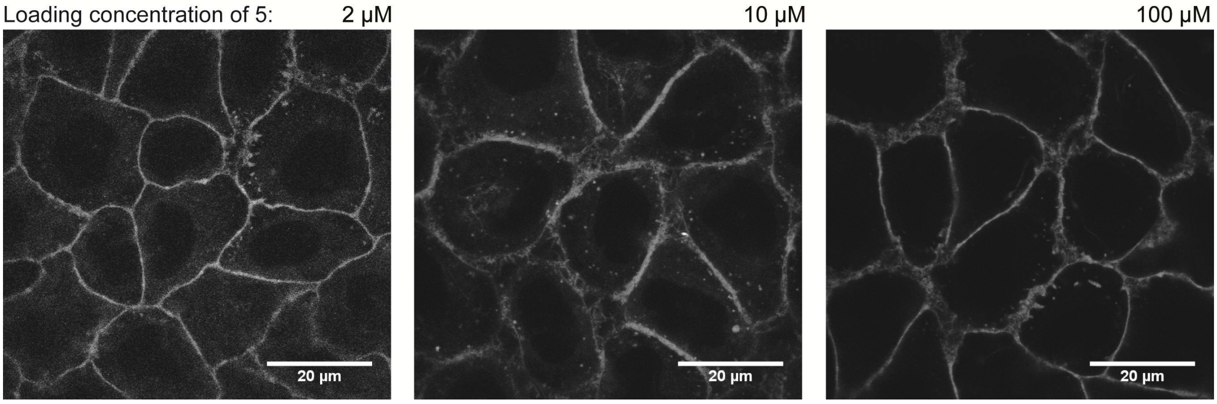


*Supplementary Figure 4. Cellular localization of* **5** *at different concentrations.* **5** stained predominantly the plasma membrane irrespective of applied loading concentration. The displayed cells are representative and the observed localization pattern did not vary across experiments. Acquisition parameters were adjusted to account for different cellular fluorophore concentrations.

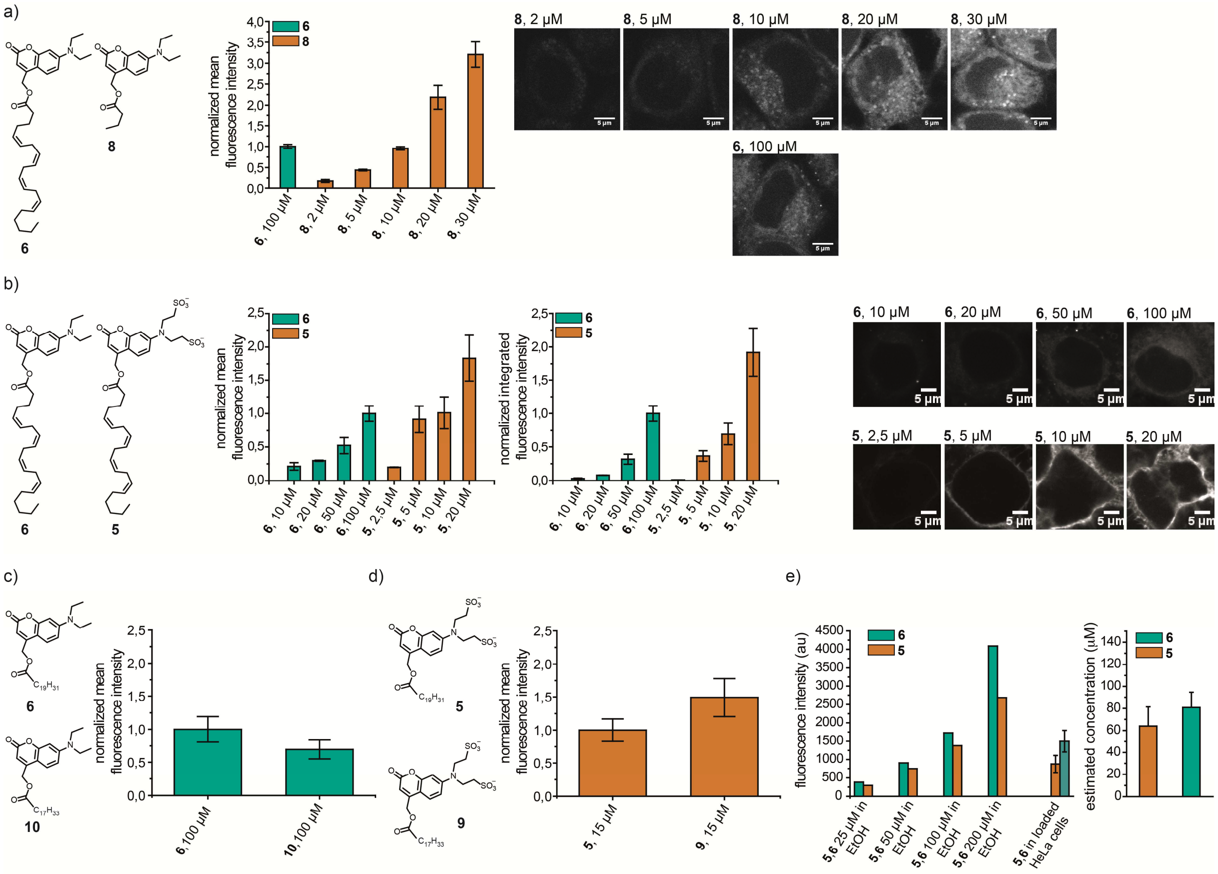


*Supplementary Figure 5. Comparison of membrane incorporation of compounds* ***5****,* ***6****,* ***8****,* ***9*** *and* ***10****.* a) Left panel: Structures of caged arachidonic acid (**6**) and butyric acid (**8**) derivatives. Middle panel: Quantification of observed coumarin fluorescence in HeLa cells after incubating with **6** or **8** at different concentrations for 20 min. Images where thresholded and the mean fluorescence intensity in the detected area measured. Values were normalized to the fluorescence intensity observed when loading HeLa cells with 100 µM **6**. Similar fluorescence intensity was observed for loading 10 µM **8** and 100 µM **6**, respectively. Compound **8** was used as a uncaging control compound in Min6 cells. To account for slight differences in uptake as compared to HeLa cells, two different concentrations were used for uncaging, 5 µM and 25 µM. No effect was observed in either case (compare Supplementary Figure 14), thereby establishing that the photoreaction as such had no influence on calcium oscillations. Right panel: Cellular localization of **6** or **8** at different concentrations. The observed localization is virtually identical, thus justifying the approach to compare the mean fluorescence intensities. Identical microscopy settings were used for image acquisition. b) Left panel: Structures of caged arachidonic acid (**6** and **5**) derivatives. Second panel to the left: Quantification of observed coumarin fluorescence in HeLa cells after incubating with **6** or **5** at different concentrations for 20 min or two minutes, respectively. Values were normalized to the fluorescence intensity observed when loading HeLa cells with 100 µM **6**. This panel displays the same analysis as for a), but the different cellular localization (right panel) is not taken into account in this analysis. In fact, the detected area for **5** is much smaller at lower concentrations, thus leading to higher mean intensity values, which do not reflect the crucial number of molecules per cell correctly. This is accounted for by comparing integrated fluorescence intensity values (the product of detected area and mean fluorescence intensity this area), as displayed in the second panel to the right. When using this approach, similar loading is observed for 100 µM **6** and 15 µM **5**, the concentrations used in uncaging experiments in MIN6 cells. c) Left panel: Structures of caged arachidonic acid and oleic acid (**6** and **10**) derivatives. Right panel: Quantification of observed coumarin fluorescence in HeLa cells after incubating with **6** or **10** (100 µM). d) Left panel: Structures of caged arachidonic acid and oleic acid (**5** and **9**) derivatives. Right panel: Quantification of observed coumarin fluorescence in HeLa cells after incubating with **6** or **10** (15 µM). e) Estimate of final concentration of caged AA derivatives after loading. Left panel: Quantification of observed coumarin fluorescence of EtOH solutions of **5** and **6** at different

concentrations and in HeLa cells using the loading conditions detailed above for both compounds. Right panel: Estimated final concentration of caged AA derivatives **5** (81±13 µM) and **6** (64±17 µM) after loading. All experiments were carried out using a Olympus FV1200 confocal microscope. Average fluorescence intensity values in the entire cell covered area were measured. Error bars represent SEM.

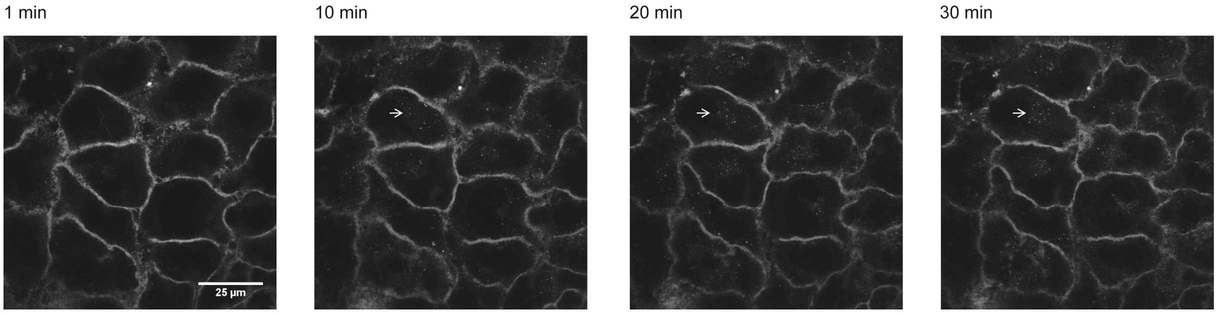


*Supplementary Figure 6. Stability of plasma membrane localization of **5**.* HeLa cells were incubated with a 10 µM solution of **5** in imaging buffer for 1 min at 25 °C, the loading solution was subsequently removed and imaging buffer added. The cells were kept at 37 °C throughout the imaging process. Images were acquired every 10 min while using identical acquisition parameters. White arrows indicate a representative region of occurring vesicles. Even though vesicles are appearing over time, the fluorescence signal very predominantly stems from plasma-membrane localized **5**. Furthermore, the detected accumulated fluorescence intensity remained relatively stable.

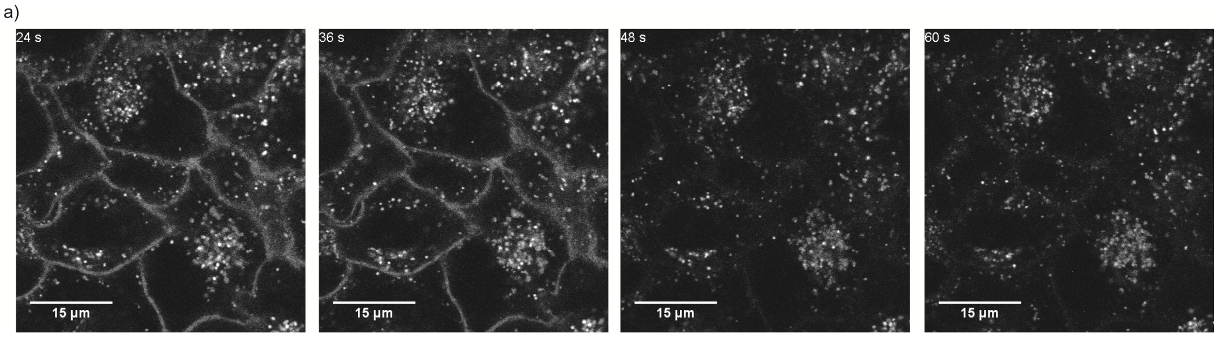


*Supplementary Figure 7. Quantification of photoreactions in living cells.* a) Time lapse montage of confocal fluorescence microscopy images acquired during a typical photoactivation experiment. HeLa cells were incubated with a 10 µM solution of **5** in imaging buffer for 2 min at 25 °C, the loading solution was subsequently removed and imaging buffer added. The cells were kept 120 min prior to imaging at 37 °C to allow for sufficient endocytosis. Images were acquired every 12 s and photoactivation (50% 405 nm laser intensity, 1 scan) was carried out between frames 36 s and 48 s. The corresponding movie M1 may be downloaded along with the SI file.

The ratio of decreases in fluorescence intensity at the plasma membrane and in vesicles $\Delta F_{(PM)}/\Delta F_{(Vesicles)}$ displayed in Figures 2d and Supplementary Figure was calculated according to the following equation:

$$\Delta F_{(PM)} \Big/ \Delta F_{(Vesicles)} = \frac{F_{0(PM)} - F_{(PM)}}{F_{0(PM)}} \Bigg/ \frac{F_{0(Vesicles)} - F_{(Vesicles)}}{F_{0(Vesicles)}} \qquad \text{(II)}$$

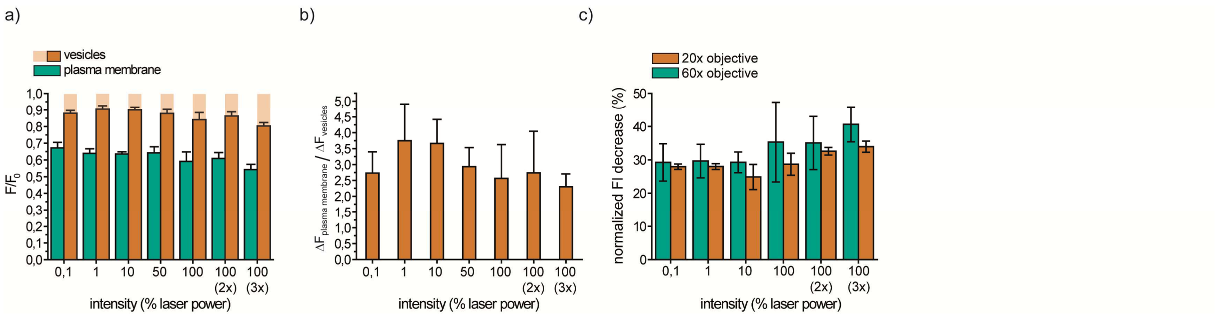


*Supplementary Figure 8. Optimization of 375 nm uncaging of compound* ***5****.* a) Remaining fluorescence intensity at the plasma membrane and in vesicular structures upon photoactivation with varied light intensity (375 nm, 2 ms pixel dwell time) (error bars represent SEM). The shaded area represents the detected fluorescence intensity in vesicular structures prior to photoactivation; the observed difference is a measure for photobleaching. b) Ratio of ΔF values determined for plasma membrane and vesicular fluorescence as a measure of photoreaction efficiency. Photoactivation at 375 nm resulted in generally higher $\Delta F_{(plasma\ membrane)}$/ $\Delta F_{(vesicles)}$ ratios as compared to 405 nm photoactivation (see Figure 2 in the main text), indicating a cleaner photoreaction and that these values are stable over a wide range of light intensities. This is not unexpected, as the absorption maximum of both coumarin species is located close to 375 nm. It was therefore decided to use 375 nm light for uncaging experiments in MIN6 cells. c) Comparison of mean fluorescence intensity decreases for uncaging through a 60x and a 20x objective with identical laser settings. Error bars represent SD. For this analysis, detected images were not segmented into ROIs featuring plasma membrane and background region (See image analysis section). Mean fluorescence intensities were measured in entire images and it was assumed that decreases in mean fluorescence intensity in the entire image are correlated with $\Delta F_{(plasma\ membrane)}$/ $\Delta F_{(vesicles)}$ ratios which can only be analyzed at higher magnification. The observed decreases in fluorescence intensity are very similar albeit slightly lower in the case of the 20x objective. This indicates that slightly higher light intensities are required for complete photoreaction in the case of the 20x objective. We therefore decided to use 3 scans (2 ms pixel dwell time) with 100% 375 nm laser intensity for uncaging experiments in MIN6 cells to ensure complete photoreaction.

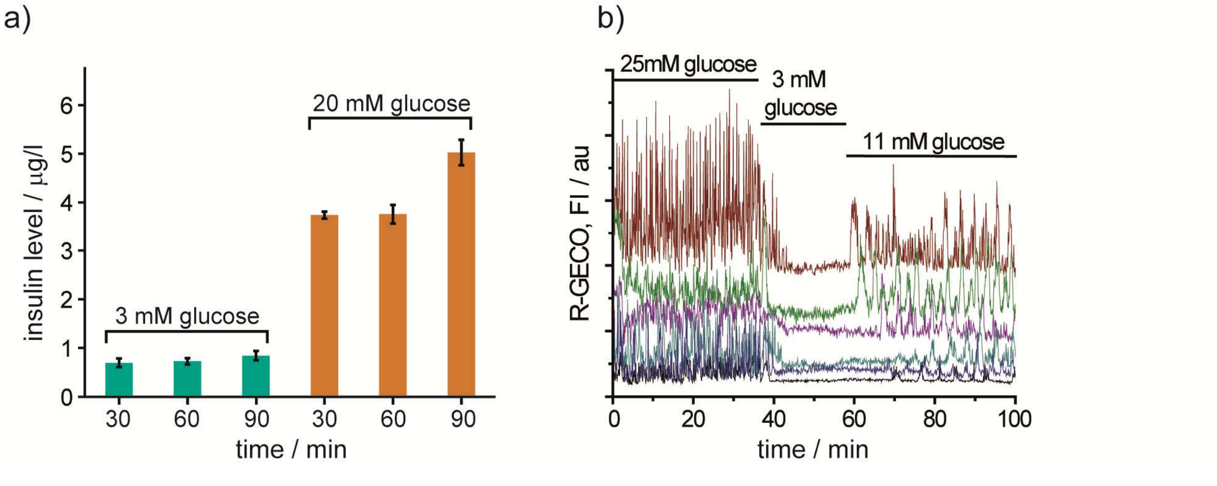


*Supplementary Figure 9. Dependence of insulin secretion and calcium oscillations of MIN6 cells on the glucose concentration in the imaging medium.* a) Dependence of insulin secretion in MIN6 cells upon glucose concentration in imaging medium (Mercodia ELISA kit 10-1247-01). Increased glucose concentration led to increased insulin secretion, thereby confirming the functional competence of the utilized cell line. Absorption was measured with a TECAN plate reader and converted to the insulin concentration according to the Mercodia kit protocol. Error bars represent SEM. b) Calcium oscillations in representative MIN6 cells at different glucose concentrations in the medium. Calcium oscillations were not observed at low glucose concentration (3 mM), occurred in a pronounced fashion at high glucose levels (20 mM) and with lower frequency and amplitude at an intermediate glucose concentration (11 mM). The cells were kept at 37 °C throughout the imaging process. Images were

acquired every 12 sec. Traces of six representative cells are displayed with suitable offsets at the y-axis for clarity.

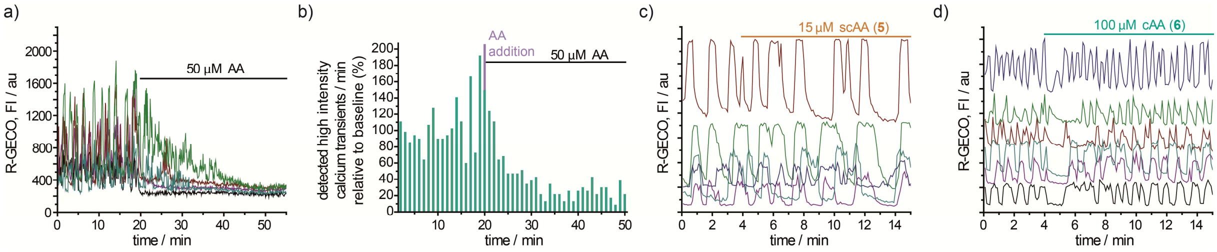


*Supplementary Figure 10. Effects of addition of free AA and caged AA derivatives* ***5*** *and* ***6*** *on calcium oscillation in glucose stimulated MIN6 cells*. The cells were kept at 37 °C throughout the imaging process. Images were acquired every 6 sec. a) Diminished calcium oscillations in MIN6 cells upon addition of free arachidonic acid (medium contained 20 mM glucose). AA addition was carried out at t = 20 min. Five representative traces are shown. b) Distribution and occurrence of detected high intensity calcium transients (>60%, per 60 s interval) in 83 MIN6 cells before and after addition of free AA (50 µM) at t = 20 min. High intensity events are less frequently observed after addition of AA, in line with diminished calcium oscillations (compare with 10a). c) Effect of addition of caged AA derivative **5** (scAA) on calcium oscillations in MIN6 cells (medium contained 20 mM glucose). Five representative traces are shown, no significant effects were observed. Traces are displayed with suitable offsets at the y-axis for clarity. d) Effect of addition of caged AA derivative **6** (cAA) on calcium oscillations in MIN6 cells (medium contained 20 mM glucose). Five representative traces are shown. A brief addition artifact is observed, as oscillations are typically diminished for 1-2 min. However, all cells resume their previous oscillation pattern after this period and no long-term effects are observed. To account for this effect, imaging for time-lapse uncaging experiments was typically started after a sufficient delay time of approximately 10 min for all compounds. Traces are displayed with suitable offsets at the y-axis for clarity.

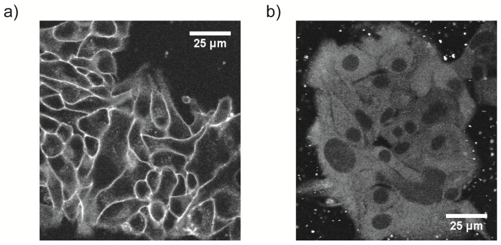


*Supplementary Figure 11. Cellular localization of compounds* ***5*** *and* ***6*** *in MIN6 cells at 37 °C.* a) Compound **5** (15 µM) predominantly stained the plasma membrane. Compound **6** (100 µM) stained internal membranes in an unspecific manner.

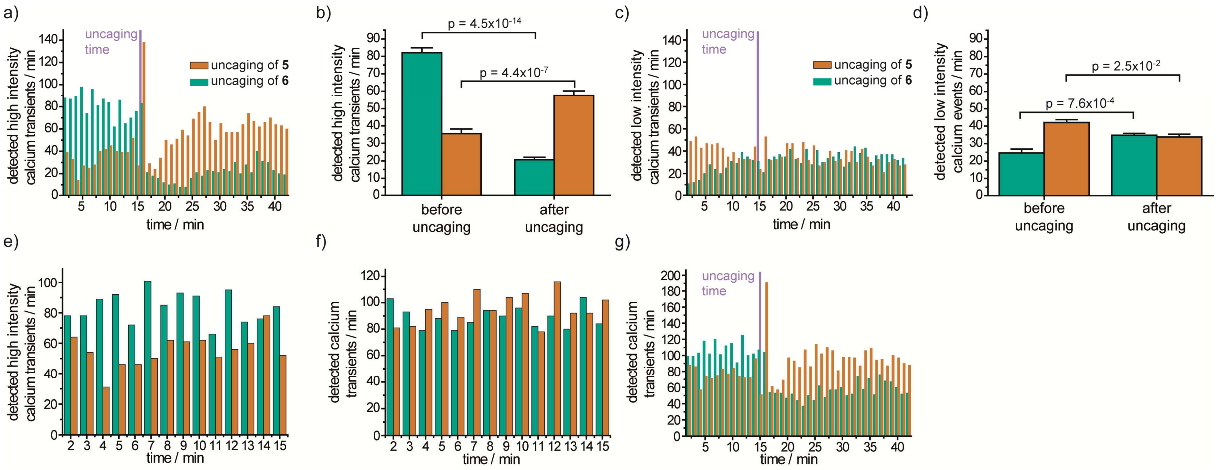


*Supplementary Figure 12. Supplementary data and alternative representations of the data displayed in Figures 3b and 3c.* a) Distribution of high intensity calcium transients, for comparison displayed in absolute numbers (detected events per 60 s interval; Figure 3b in the main text). b) Average numbers of detected high intensity calcium events per 60 s interval before and after uncaging AA from compounds **5** and **6**. c) Distribution of low intensity calcium events, displayed in absolute numbers. d) Average numbers of detected low intensity calcium events per 60 s interval before and after uncaging AA from compounds **5** and **6**. e) Distribution of high intensity calcium events prior to uncaging; in this case the peak detection algorithm was used only to detect calcium events prior to uncaging AA from either **5** or **6**. f) Distribution of all detected intensity calcium events prior to uncaging; in this case the peak detection algorithm was used only to detect calcium events prior to uncaging AA from either **5** or **6**. g) Distribution of all detected calcium events, displayed in absolute numbers (detected events per 60 s interval). Error bars represent SEM.

Discussion of displayed data and choice of threshold criteria

*Note: The dataset discussed in detail here is displayed in figures 3b-d in the main text (AA uncaging from compounds **5** and **6**), all other datasets were analyzed using identical image analysis parameters and threshold criteria.*

*Peak-detection approach:* More high intensity events were detected prior to uncaging **6** than prior to uncaging **5** (Supplementary Figure 12a). This is, however, partly due to the internal normalization which is required to define high intensity events. As uncaging AA at the plasma membrane (from **5**) leads to augmented calcium oscillations, the highest detected calcium event in theses traces which serves as reference value for the internal normalization is usually located in the post-uncaging portion of the trace. Consequently, pre-uncaging events are more likely to be classified as “low-intensity”. The opposite holds true for uncaging AA at the plasma membrane (from **6**) as oscillations are typically diminished in this case. This effect can be quantified by conducting the peak-detection analysis only in the pre-uncaging section, where no distinctly different calcium patterns are observed (displayed in Supplementary Figure 12e and Supplementary Figure 12f). The difference in detected high intensity events is much smaller in this case (Supplementary Figure 12e, as compared to Supplementary Figure 12a) and the overall number of detected events is virtually identical (Supplementary Figure 12f), indicating very similar pre-uncaging calcium dynamics. These observations highlight that this statistical approach is very powerful in detecting relative changes in dynamic signaling patterns, but that comparisons of absolute count numbers from different groups can lead to misinterpretation of data if different perturbations of the system under investigation cause significantly different overall signaling patterns. Consequently, this study does not rely on comparing absolute count numbers for its conclusions.

*Threshold criterion for distinguishing high and low intensity $Ca^{2+}$ events*: An automatic peak detection routine had to be employed as the acquired data set was relatively large (~ 6800 individual calcium transients). Taking into account that non-uniform expression levels of the employed genetically encoded fluorescent calcium sensor R-GECO resulted in a broad distribution of fluorescence intensity

levels, the detection of false positives (small maxima in the traces which narrowly exceeded the internal threshold of the algorithm, but presumably were not indicative of actual changes in calcium concentrations) was unavoidable. To account for this, we divided the detected events into high intensity events, which represent true changes in calcium concentration and low intensity events, which are heavily compromised by random fluctuations in the acquired time traces. Applying the described 60% threshold appeared be a reasonable solution. Neither uncaging of AA from **5** nor **6** had a major effect on the occurrence of low intensity events (Supplementary Figure 12c and Supplementary Figure 12d) when compared to the effects observed for high intensity events (Figures 3a and 3b in the main text and Supplementary Figure 12a and Supplementary Figure 12b). This finding is in line with the notion that low intensity events mainly represent statistical noise which should not be affected by the uncaging assay. The overall short- and long-term changes in calcium dynamics upon uncaging AA from compounds **5** and **6** may still be appreciated in the distribution of all detected calcium events (Supplementary Figure 12g), albeit with a higher baseline due to the presence of low intensity events. Supplementary Figures 12c, 12d, and 12f were included to provide the reader with the entire dataset; however, we refrained from drawing any conclusions from the distribution of low intensity events. All conclusions made in this study are solely based on the distribution of high intensity calcium events.

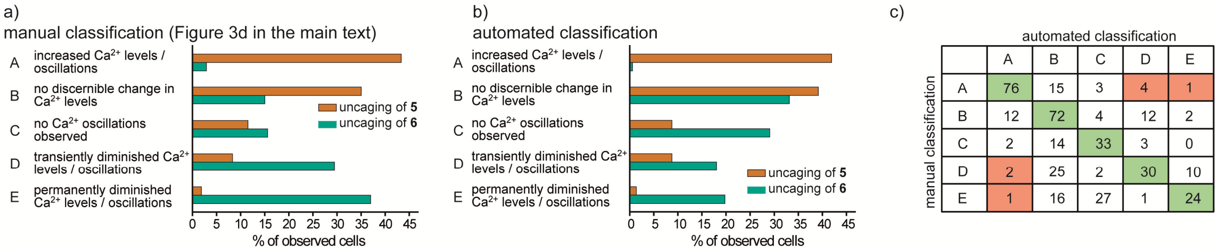


*Supplementary Figure 13. Automated classification of cellular response patterns upon uncaging of compounds **5** and **6**.* a) Results of manual classification, for comparison (Figure 3e in the main text). b) Results of automated classification. c) Correlation of automated and manual classification. Green: Cells which were classified in the same categories. Red: Cells which were classified in opposing categories.

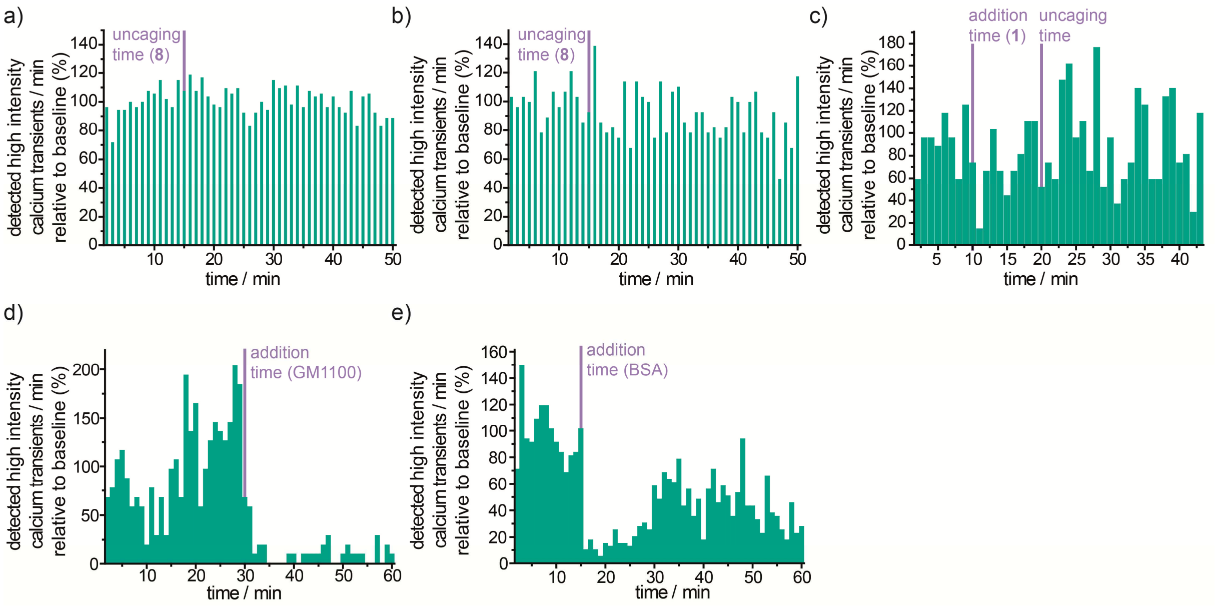


*Supplementary Figure 14. Control experiments for data shown in figures 3 and 4 in the main text.* a) Uncaging of **8** (caged butyrate), 5 µM loading concentration, uncaging time at t= 10 min b) Uncaging of **8**, 25 µM loading concentration, uncaging time at t= 10 min. The distribution and occurrence of detected high intensity calcium events was not significantly altered in both cases. For cellular uptake rates of **c**ompound **8** please compare with Supplementary Figure 5. c) Cellular responses to addition and irradiation of compound **1** (10 µM) in MIN6 cells. The occurrence and distribution of detected high intensity calcium events was not significantly altered in both cases. d) Occurrence of detected high intensity calcium events within every interval of 60 s before and after addition of the specific GPR40 inhibitor GM1100 (10 µM). Occurrence of detected high intensity calcium events within every interval of 60 s before and after addition of bovine serum albumin (125 µM).

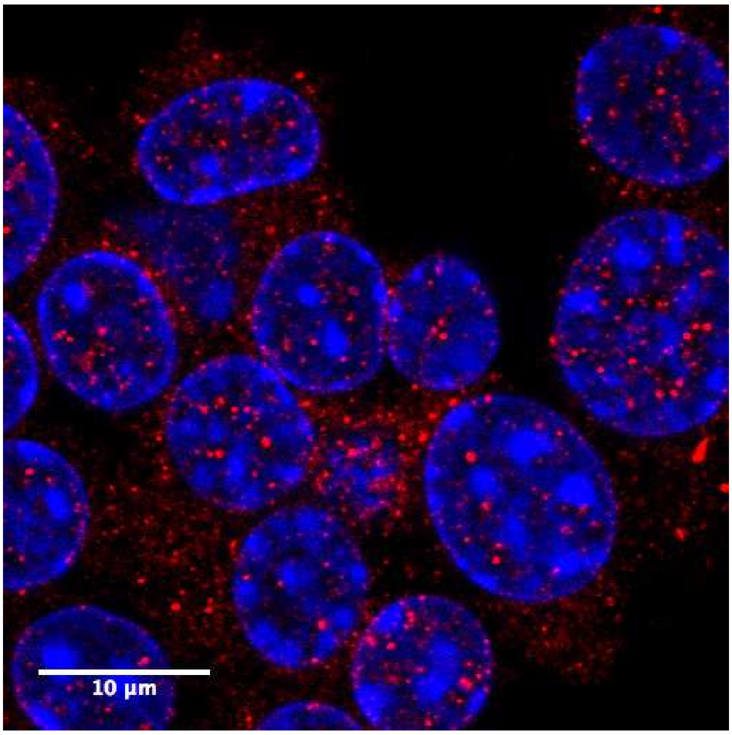


*Supplementary Figure 15.* Immunostaining of GPR40 in MIN6 cells. Glucose stimulated MIN6 cells were fixed with 4%PFA and stained with anti-GPR40 (mouse, developed in rabbit) primary antibody (Innovagen, PA-2210-100) and goat anti-rabbit 488 secondary antibody (Lifetechnology, A11008) with subsequent staining with Hoechst dye.

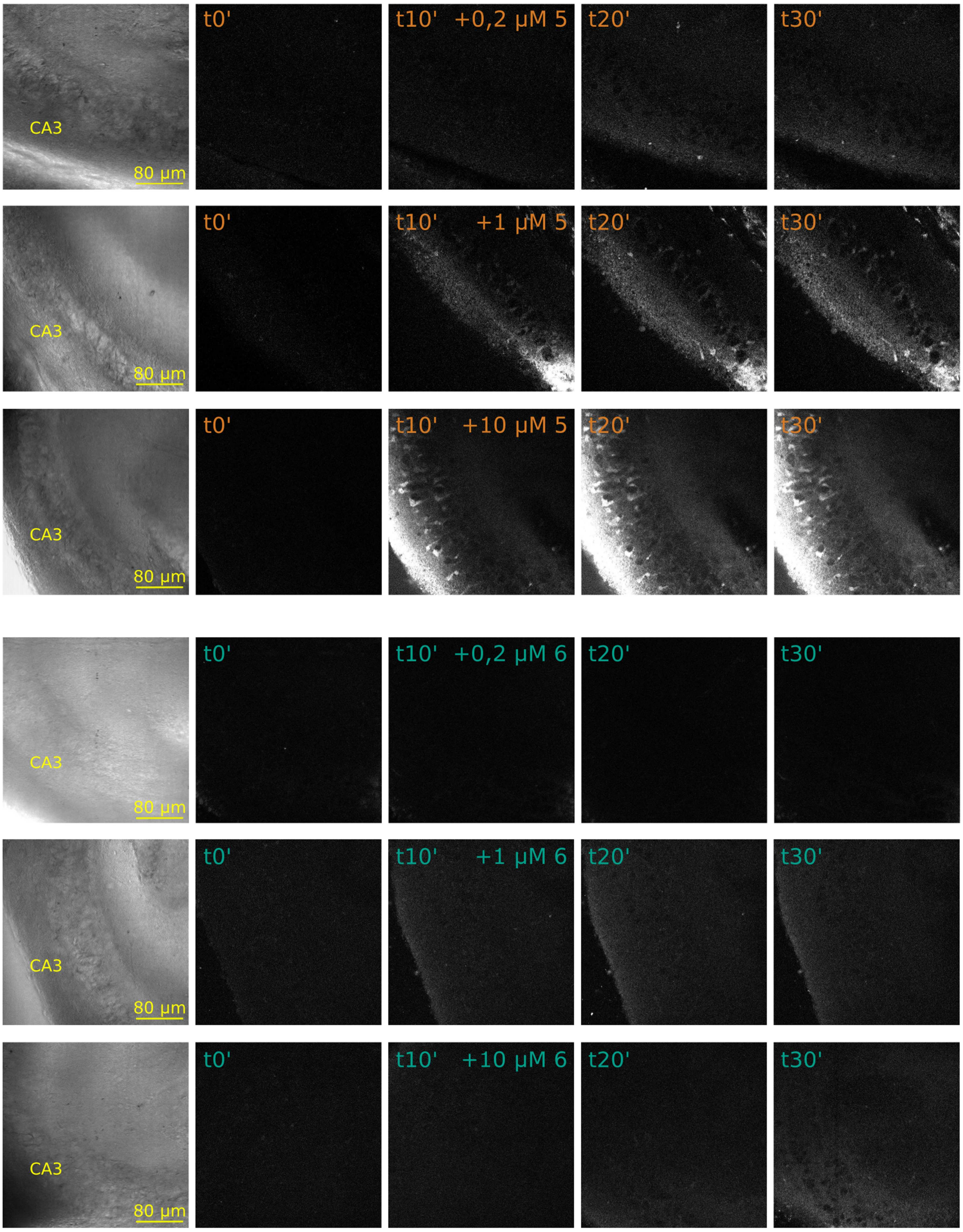


*Supplementary Figure 16. Coumarin fluorescence detected in mouse brain slices.* Images were acquired using two-photon imaging. The excitation wavelength was 720 nm; coumarin fluorescence was recorded at 500 nm. Images were aquired with a 25X, 0.95 N.A. water immersion objective, at a depth of 40 µm from the slice surface, a location at which CA3 pyramidal cells where typically patched in electrophysiological experiments. Experiments were performed at ~ 25ºC using standard oxygenated ACSF. On the left column, transmission images of the CA3 region of acute hippocampal slices. Two-photon images were acquired before the addition of the caged compound (t0') and every 10 minutes for 30 minutes. Similar distributions of coumarin fluorescence were observed in a second set of experiments.

# Supplementary Notes

*Supplementary Note 1. Chemical synthesis and analytical data of new compounds*

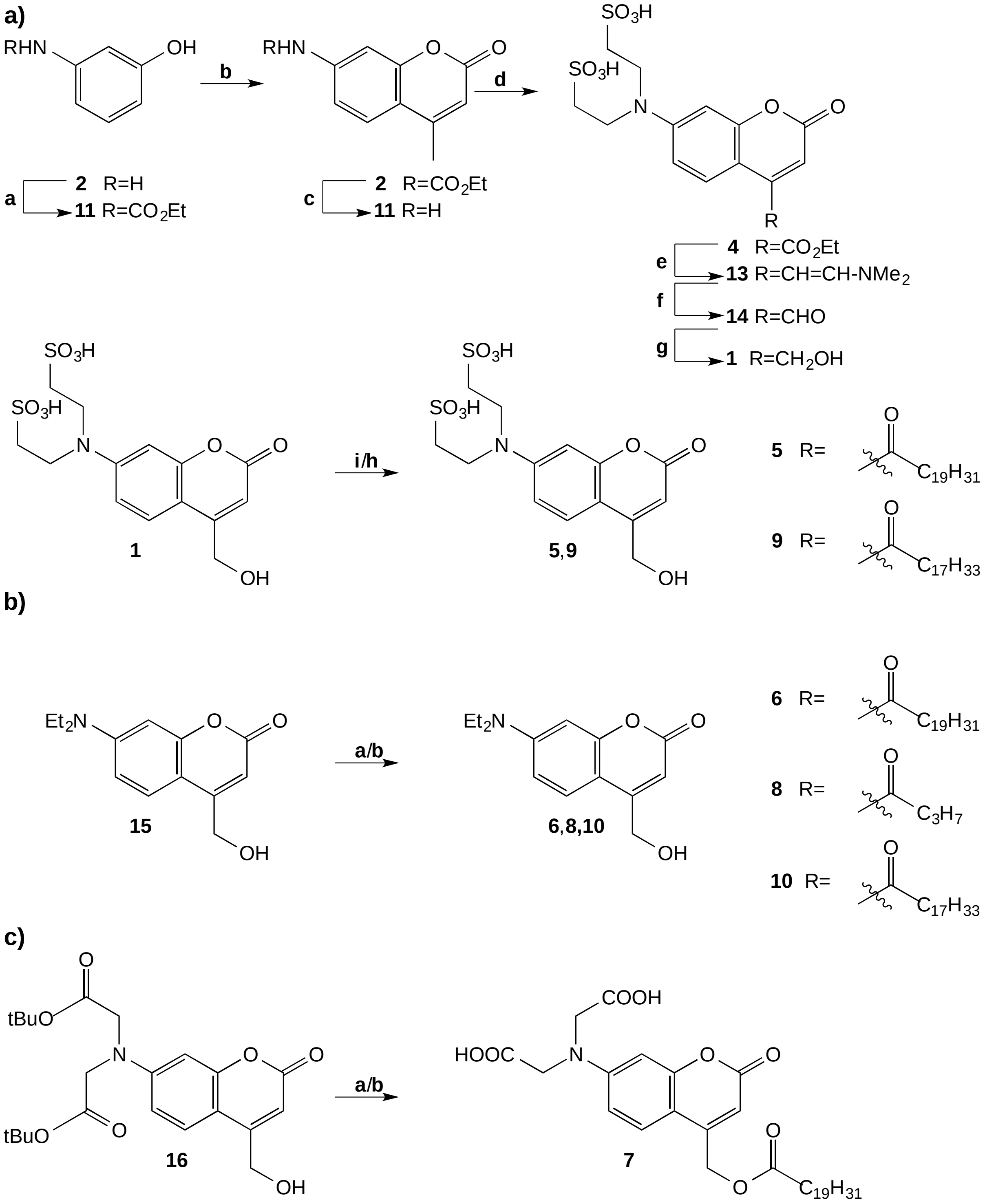


Scheme S1: Synthesis of compounds **5-10**. a) Reagents and conditions: **a** $ClCO_2Et$, pyridine, THF, 15 h, 3-20°C, 80%; **b** $CH_3COCH_2CO_2Et$, 77% $H_2SO_4$, 1.5 h, 21 °C, 91%; **c** $H_2SO_4$:AcOH 1:1, 2.5 h, 125-130°C, 80%; **d** $BrCH_2CH_2SO_3Na$, NaI, *i*-$Pr_2NEt$, DMF, 120°C, 40 h, 64%; **e** $Me_2N$-$CH(OMe)_2$, DMF, 22 h, 140 °C; **f** $NaIO_4$, THF:$H_2O$ 1:1, 3 h, 20 °C; **g** $NaBH_4$, methanol, 3 h, 20 °C, 32% over three steps; **h** arachidonic acid, EDC*HCl, DMAP, DMF, 22 h, 21 °C, 88%; **i** oleic acid, EDC*HCl, DMAP, DMF, 22 h, 21 °C, 94%. b) Reagents and conditions: **a** arachidonic acid, EDC*HCl, DMAP,

DCM, 6 h, 21 °C, 80 %; **b** butyric acid, EDC*HCl, DMAP, DCM, 6 h, 21 °C, 95 %. **c** oleic acid, EDC*HCl, DMAP, DCM, 6 h, 21 °C, 95 %. c) **a** arachidonic acid, EDC*HCl, DMAP, DCM, 6 h, 21 °C, 65 %; **b** 95 % TFA, 0 °C.

THF: tetrahydrofuran, DMF *N,N*-dimethylformamide, EDC*HCl *N*-(3-dimethylaminopropyl)-*N'*-ethylcarbodiimide hydrochloride, DMAP 4-(*N,N*-dimethylamino)pyridine, DCM: dichlormethan, TFA: trifluoroacetic acid.

General synthetic procedures and description of conducted syntheses

All chemicals were obtained from commercial sources (Acros, Sigma, Aldrich, Enzo, Lancaster or Merck) and were used without further purification. Solvents for flash chromatography were obtained from VWR and dry solvents were obtained from Sigma. Deuterated solvents were obtained from Deutero GmbH, Karlsruhe, Germany. All reactions were carried out using dry solvents under an inert atmosphere unless otherwise stated in the respective experimental procedure. TLC was performed on precoated plates of silica gel (Merck, 60 F254) using UV light (254 or 366 nm) or a solution of phosphomolybdic acid in EtOH (10 g phosphomolybdic acid, in 100 mL EtOH) for analysis. Preparative column chromatography was performed using silica form Merck, Darmstadt, Germany (silica 60, grain size 0.063-0.200 mm) with a pressure of 1 - 1.5 bar. HPLC was performed on a Knauer HPLC Smartline Pump 1000 using a Knauer Smartling UV Detector 2500 instrument. A 250x10 mm LiChrospher 100 RP-18 column was used for semi-preparative HPLC applications. $^{1}$H- and $^{13}$C-NMR-spectra were obtained on a 400 MHz Bruker UltraShield$^{TM}$ spectrometer. Chemical shifts of $^{1}$H- and $^{13}$C-NMR-spectra are referenced indirectly to tetramethylsilane. *J* values are given in Hz and chemical shifts in ppm. Splitting patterns are designated as follows: s, singlet; d, doublet; t, triplet; q, quartett; m, multiplet; b, broad. $^{13}$C-NMR-spectra were broadband hydrogen decoupled. Mass spectra (ESI) were recorded using a Waters Micromass ZQ massspectrometer or a HP Esquire-LC mass spectrometer. High-resolution mass spectra were recorded at the University of Heidelberg.

N-ethylcarbamoyl-3-aminophenol **11**was condensed with ethyl acetoacetate in the presence of sulfuric acid to afford coumarin precursor **3** which, after deprotection with 1:1 sulfuric acid:glacial acetic acid mixture was alkylated with sodium 2-bromoethanesulfonate in *N,N*-dimethylformamide using *N,N*-di*iso*propylethylamine as proton scavenger. The bis-sulfonated, water soluble 7-amino-4-methylcoumarin derivative **4** was purified by reverse phase chromatography employing a triethylammonium bicarbonate buffer system and obtained as its bis-triethylammonium salt. The 4-methyl group was converted to the alcohol using a previously reported protocol in three steps. Esters were prepared from the alcohol in a carbodiimide mediated reaction in the presence of 4-*N,N*-dimethylaminopyridine utilizing arachidonic acid or oleic acid to give compound **5** and **9**, respectively. The target compounds were obtained as readily soluble triethylammonium salts from reverse phase chromatography. Coumarin precursors **15** and **16** were synthesized according to literature protocols. They were coupled to the respective carboxylic acids using carbodiimide mediated reactions in the presence of 4-*N,N*-dimethylaminopyridine which yielded coumarin esters **6**, **8**, **10** and **17**. The synthesis of **6** was published previously by our group. The $^{t}$Bu protections groups of **17** were removed by treatment with trifluoracetic acid. For references see main text.

Ethyl 3-(*N*-carbamoyl)aminophenol **11**

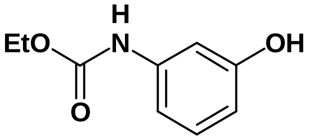

Ethyl chloroformate (87.3 mL, 0.92 mol) was slowly added to a solution of 3-aminophenol **2** (100 g, 0.92 mmol) in anhydrous tetrahydrofuran (100 mL) and dry pyridine (88.9 mL, 1.1 mol) at below 10 °C with stirring under argon during 2.5 h and cooling in an ice bath. The cooling bath was removed and the mixture was stirred 18 h at 30 °C. The mixture was diluted with dichloromethane (300 mL), washed four times with water (4 x 300 mL), dried ($Na_2SO_4$), filtered and concentrated under reduced pressure. The black oil obtained (200 g) was diluted with toluene (50 mL) and cooled in an ice bath. The white precipitate was collected, washed with toluene (100 mL) and hexane (50 mL) and dried to afford pure ethyl 3-(*N*-carbamoyl)aminophenol (85.5 g, 51.5%) as colorless crystals. Additional product was obtained from the mother liquor by repeated crystallizations.

Overall yield 132.7 g (79.9%).

$^1$H NMR (400 MHz, DMSO) δ = 9.44 (s, 1H), 9.30 (s, 1H), 7.07 – 6.98 (m, 2H), 6.86 (d, *J* = 9.1 Hz, 1H), 6.39 (dd, *J* = 8.1, 1.4 Hz, 1H), 4.11 (q, *J* = 7.1 Hz, 2H), 1.24 (t, *J* = 7.1 Hz, 3H).

$^{13}$C NMR (101 MHz, DMSO) δ = 158.14, 153.89, 140.74, 129.76, 109.91, 109.51, 105.83, 60.45, 40.62, 40.41, 40.20, 39.99, 39.78, 39.57, 39.36, 14.98.

Ethyl 4-methyl-7-*N*-carbamoyl-2-oxo-2*H*-chromen **3**

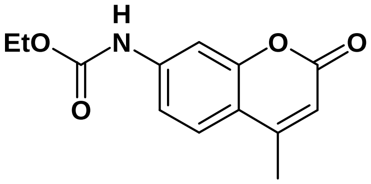

A solution of **11** (19.26 g, 106.3 mmol), ethyl acetoacetate (16.1 mL, 127.6 mmol) and ice cold 77% sulfuric was stirred at 20 °C for 1.5 h and poured onto crushed ice (400 mL). The white precipitate was filtered off and dried in high vacuum. The title compound was obtained as a colorless solid (24 g, 91%).

$^1$H NMR (400 MHz, DMSO) δ = 10.13 (s, 1H), 7.66 (d, *J* = 8.7 Hz, 1H), 7.52 (d, *J* = 2.0 Hz, 1H), 7.38 (dd, *J* = 8.7, 2.1 Hz, 1H), 6.21 (d, *J* = 1.2 Hz, 1H), 4.15 (q, *J* = 7.1 Hz, 2H), 2.38 (d, *J* = 1.2 Hz, 3H), 1.24 (t, *J* = 7.1 Hz, 3H).

$^{13}$C NMR (101 MHz, DMSO) δ = 160.51, 154.31, 153.80, 153.64, 143.34, 126.42, 114.73, 114.70, 112.30, 104.85, 61.16, 18.43, 14.86.

7-Amino-4-methyl-2-oxo-2*H*-chromen **12**

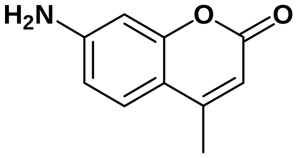

Compound **3** (24 g, 97 mmol) was suspended in a mixture of acetic acid (20 mL, 350 mL) and concentrated sulfuric acid (20 mL, 373 mmol, 95-97%) the reaction mixture stirred at 125-135 °C. The carbamate soon dissolved. After 2.5 h an orange-yellow precipitate was formed. The slurry was allowed to cool and poured onto crushed ice (600 mL). The pH was adjusted to 7 by addition of ice-cold 10% sodium hydroxide solution (from 30 g NaOH and 270 mL water) and 1 N NaOH. Finally, the pH was brought to 12 (final volume was about 1 L). The precipitate was filtered off, washed with

water (250 mL) and dried in high vacuum overnight (desiccator). The title compound was obtained as tan colored solid (13.6 g, 80%)

$^{1}$H NMR (400 MHz, DMSO) δ = 7.38 (d, *J* = 8.6 Hz, 1H), 6.54 (dd, *J* = 8.6, 2.1 Hz, 1H), 6.38 (d, *J* = 2.1 Hz, 1H), 6.09 (s, 2H), 5.88 (s, 1H), 2.28 (s, 3H).

$^{13}$C NMR (101 MHz, DMSO) δ = 161.24, 155.90, 154.24, 153.50, 126.65, 111.64, 109.32, 107.92, 98.99, 18.46.

7-Amino-4-methyl-2-oxo-2*H*-chromen *N*,*N*-bis-ethanesulfonic acid **4**

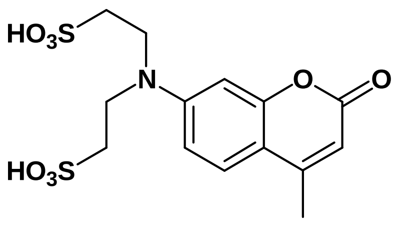


A slurry of **12** (1.55 g, 8.8 mmol), sodium iodide (150 mg, 1 mmol), di*iso*propylethylamine (8.6 mL, 50 mmol) and sodium 2-bromoethanesulfonate (9.5 g, 45 mmol) in *N*,*N*-dimethylformamide (20 mL) was vigorously stirred at 120 °C under argon. After 40 h heating was terminated and the mixture was allowed to cool to room temperature. Volatiles were removed under high vacuum at 50 °C. The remaining slurry was suspended in boiling ethanol (60 mL), cooled to room temperature and filtered. The filter cake was washed with ethanol (50 mL) and subjected to chromatography on a column of Oligoprep RP18 (100 g) with an eluent containing 50 mM triethylammonium bicarbonate and increasing methanol concentration (1. 0% methanol, 750 mL; 2. 10% methanol, 500 mL; 3. 20% methanol, 500 mL, 4. 40% methanol, 500 mL) to afford the title compound as triethylammonium salt (3.35 g, 64%) as a hydroscopic yellowish solid.

HPLC: $t_R$ 30% methanol, 50 mM triethylammonium formate = 5.3 min.

$^{1}$H NMR (400 MHz, $CDCl_3$) δ = 7.43 (d, *J*=8.9, 1H), 6.82 (dd, *J*=9.0, 2.5, 1H), 6.75 (d, *J*=2.5, 1H), 5.95 (d, *J*=0.8, 1H), 3.92 (m, 4H), 3.15 (q, *J*=7.3, 12H), 3.12 (m, 4 H), 2.36 (d, *J*=0.8, 3H), 1.39 (t, *J*=7.3, 18H).

$^{13}$C NMR (101 MHz, $CDCl_3$) δ = 161.99, 155.77, 153.23, 150.10, 125.90, 109.75, 109.05, 97.97, 48.14, 47.40, 46.38, 18.45, 8.97.

HR-MS (ESI negative) m/z found 390.03176, calculated for $C_{14}H_{16}NO_8S_2$ 390.03228 $[M-H]^-$.

The mono-sulfonated derivative was found to be the major byproduct and also isolated and characterized.

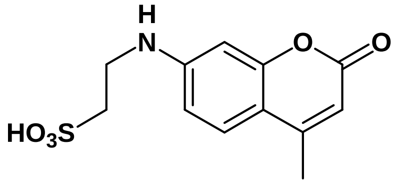


HPLC: $t_R$ 30% methanol, 50 mM triethylammonium formate = 12.5 min.

$^{1}$H NMR (400 MHz, $CDCl_3$) δ = 7.28 (d, *J*=8.7, 1H), 6.52 (dd, *J*=8.7, 2.1, 1H), 6.38 (d, *J*=2.1, 1H), 5.89 (s, 1H), 5.72 (t, *J*=4.8, 1H, NH), 3.62 – 3.49 (m, 2H), 3.14 (q, *J*=7.2, 6H), 3.13 -3.03 (m, 2H), 2.28 (s, 3H), 1.32 (t, *J*=7.3, 9H).

$^{13}C$ NMR (101 MHz, $CDCl_3$) δ = 162.16, 155.88, 153.40, 151.67, 125.46, 110.87, 110.23, 108.86, 97.63, 49.53, 46.38, 39.52, 18.53, 8.70.

HR-MS (ESI negative) m/z found 282.04369, calculated for $C_{12}H_{12}NO_5S$ 282.04417 $[M-H]^-$.

7-Amino-4-hydroxymethyl-2-oxo-2*H*-chromen-*N*,*N*-bis-ethanesulfonic acid **1**

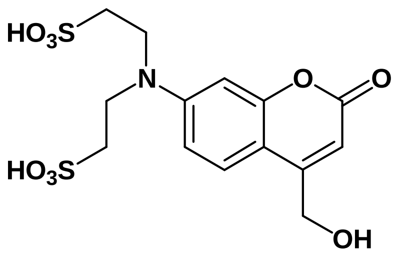


A solution of **4** (3.34 g, 5.6 mmol) in anhydrous *N,N*-dimethylformamide (3 mL) and *N,N*-dimethylformamide dimethylacetal (7 mL, 49.5 mmol) was heated to 140 °C for 22 h under argon. After cooling to room temperature, volatiles were removed under high vacuum. The crude compound **13** was directly used in the next step. A solution of crude **13** in tetrahydrofuran:$H_2O$ (1:1, 30 mL) was treated with sodium periodate (2.7 g, 12.6 mmol) at 20 °C with vigorous stirring. After 3 h the mixture was filtered and the filtrate evaporated under reduced pressure to afford crude compound **14** which was directly subjected to the next transformation. A suspension of crude **14** in methanol (20 mL) was treated with sodium borohydride (3.6 g, 95.1 mmol) portion wise with stirring at 20 °C. After 2 h, analytical HPLC (50 mM triethylammonium formate, 30% methanol, pH 6, $t_R$ **14** = 2.1 min) indicated complete conversion of the starting material. The mixture was neutralized with concentrated hydrochloric acid and the solvent removed under reduced pressure. The obtained residue was suspended in methanol (50 mL) and filtered. The filter cake was washed with methanol and allowed to dry in the hood to give a brown solid (4.6 g). The filtrate was evaporated to yield a brown semi-solid (3 g). This material was subjected to flash chromatography purification on a LiChroprep RP18 column (40-63 µm, 16% C, 12 x 4 cm) with 100 mM triethylammonium bicarbonate buffer containing 1-20% methanol (with a stepwise gradient of ~250 mL). The filter cake was purified likewise. Fractions from above runs containing the pure compound were combined and the solvent removed under reduced pressure.

Impure fractions were combined and, after removal of volatiles under reduced pressure, again subjected to purification.

Yield: 1.1 g (32% based on the amount of **4** used, over three steps), hygroscopic solid

HPLC: $t_R$ 20% methanol 50 mM triethylammonium formate = 5.4 min;

$^1H$ NMR (400 MHz, DMSO) δ = 7.45 (d, *J* = 9.0 Hz, 1H, H-5), 6.65 (dd, *J* = 9.0, 2.3 Hz, 1H, H-6), 6.51 (d, *J* = 2.3 Hz, 1H, H-8), 6.07 (s, 1H, H-3), 4.66 (s, 2H, $CH_2$-O), 3.61 (m, 4H), 2.90 (q, *J* = 7.3 Hz, 20H), 2.69 (m, 4H), 1.09 (t, *J* = 7.2 Hz, 30H).

$^{13}C$ NMR (101 MHz, DMSO) δ = 161.55, 157.41, 155.88, 150.39, 125.63, 109.01, 106.62, 104.70, 97.49, 59.46, 48.34, 47.87, 46.18, 9.86.

HR-MS (ESI negative) m/z found 406.02677, calculated for $C_{14}H_{16}NO_9S_2$ 406.02720 $[M-H]^-$.

(7-*N*,*N*-Di(2-sulfonylethyl)amino)-2-oxo-2*H*-chromen-4-yl)methyl arachidonate **5**

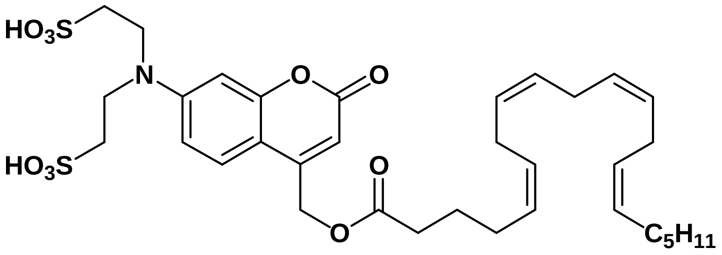

A solution of **1** (110 mg, 180 µmol $NEt_3$-salt) in anhydrous *N*,*N*-dimethylformamide (1 mL) was evaporated at 0.2 mbar. Under argon atmosphere, the residue was dissolved in *N*,*N*-dimethylformamide (1 mL) and arachidonic acid (66 µL, 198 µmol), *N*-(3-dimethylaminopropyl)-*N'*-ethylcarbodiimide hydrochloride (38 mg, 198 µmol) and 4-(*N*,*N*-dimethylamino)pyridine (10 mg, 82 µmol) were subsequently added. The flask was tightly sealed and the mixture was stirred in the dark. After 22 h volatiles were evaporated at 0.2 mbar. The residue was purified by flash chromatography on a column of LiChroprep RP18, 40-63 µm, 16% C (92 g, 14 x 3 cm) with 1. 25 mM triethylammonium bicarbonate, 50% methanol (600 mL) and 2. 50 mM triethylammonium bicarbonate, 94% methanol (~200 mL). The title compound was obtained as brightly yellow oil after removing the solvents under reduced pressure (142 mg, 88%).

HPLC: $t_R$ (90% methanol 50 mM triethylammonium formate pH 6) = 3.2 min.

$^1$H NMR (400 MHz, $CDCl_3$) δ = 10.01 (s, 2H, 2 x $NH^+$), 7.30 (d, *J*=9.0, 1H, H-5), 6.76 (dd, *J*=9.0, 2.4, 1H, H-6), 6.72 (d, *J*=2.4, 1H, H-8), 6.09 (s, 1H, H-3), 5.44 – 5.25 (m, 8H), 5.19 (s, 2H, $CH_2$-O), 3.97 – 3.82 (m, 4H), 3.28 – 3.13 (m, 12H), 3.13 – 3.02 (m, 4H), 2.87 – 2.70 (m, 8H), 2.43 (t, *J*=7.6, 2H), 2.30 (t, *J*=7.6, 1H), 2.18 – 2.06 (m, 3H), 2.02 (q, *J*=6.9, 3H), 1.71 (dq, *J*=23.2, 7.5, 3H), 1.37 (t, *J*=7.3, 18H), 1.34 – 1.15 (m, 10H), 0.85 (t, *J*=6.8, 3H, $CH_3$).

$^{13}$C NMR (101 MHz, $CDCl_3$) δ = 172.76, 161.67, 156.06, 150.36, 149.77, 130.47, 129.12, 128.65, 128.57, 128.26, 128.07, 127.85, 127.53, 124.77, 109.41, 106.96, 106.80, 98.39, 61.19, 48.15, 47.48, 46.38, 33.43, 31.48, 29.29, 27.19, 26.51, 25.62, 24.66, 22.53, 14.04, 8.73.

HR-MS (ESI negative) m/z found 692.25702, calculated for $C_{34}H_{46}NO_{10}S_2$ 692.25686 $[M-H]^-$.

(7-*N*,*N*-Di(2-sulfonylethyl)amino)-2-oxo-2*H*-chromen-4-yl)methyl oleate **9**

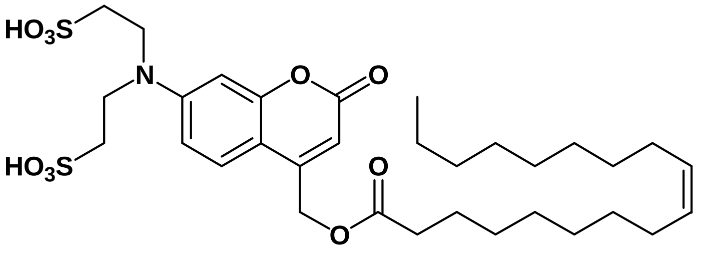

A solution of **1** (100 mg, 141 µmol $NEt_3$-salt) in *N*,*N*-dimethylformamide (1 mL) was evaporated at 0.3 mbar. Under argon atmosphere, the residue was dissolved in *N*,*N*-dimethylformamide (1 mL) and oleic acid (57.3 µL, 180 µmol), *N*-(3-dimethylaminopropyl)-*N'*-ethylcarbodiimide hydrochloride (34.6 mg, 180 µmol) and 4-(*N*,*N*-dimethylamino)pyridine (10 mg, 82 µmol) were subsequently added. The flask was tightly sealed and the mixture was stirred in the dark. After 22 h volatiles are evaporated at 0.3 mbar. The residue was purified by flash chromatography on a column of LiChroprep RP18, 40-63 µm, 16% C (92 g, 14 x 3 cm) with 1. 25 mM triethylammonium bicarbonate, 50% methanol (500 mL) and 2. 50 mM triethylammonium bicarbonate, 94% methanol (~250 mL). The title compound was obtained as brightly yellow oil after removing the solvents under reduced pressure.

Yield: 92 mg (74.8%) after drying at 0.004 mbar for 2 h,

HPLC: $t_R$ (90% methanol 50 mM triethylammonium formate pH 6) = 5.4 min.

$^1$H NMR (400 MHz, $CDCl_3$) δ = 10.01 (bs, 2H, 2xNH$^+$), 7.30 (d, *J* = 9.0 Hz, 1H, H-5), 6.76 (dd, *J* = 9.0, 2.4 Hz, 1H, H-6), 6.71 (d, *J* = 2.4 Hz, 1H, H-8), 6.09 (s, 1H, H-3), 5.31 (m, 2H), 5.19 (s, 2H, $CH_2O$), 3.88 (m, 4H), 3.18 (qd, *J* = 7.3, 4.9 Hz, 12H), 3.08 (m, 4H), 2.41 (t, *J* = 7.6 Hz, 2H, $CH_2$), 1.98 (m, 4H), 1.65 (m, 2H, $CH_2$), 1.37 (t, *J* = 7.3 Hz, 18H), 1.26 (m, 20H), 0.84 (t, *J* = 6.8 Hz, 3H, $CH_3$).

$^{13}$C NMR (101 MHz, $CDCl_3$) δ = 173.03, 161.71, 156.01, 150.25, 149.86, 129.99, 129.73, 124.79, 109.39, 106.89, 106.82, 98.36, 61.13, 48.10, 47.47, 46.34, 34.07, 31.88, 29.74, 29.66, 29.50, 29.29, 29.13, 29.11, 29.08, 27.19, 27.14, 24.82, 22.66, 14.11, 8.73.

HR-MS (ESI negative) m/z found 670.27171, calculated for $C_{32}H_{48}NO_{10}S_2$ 670.27251 $[M-H]^-$.

(7-*N*,*N*-Diethylamino)-2-oxo-2*H*-chromen-4-yl)methyl butyrate (**8**)

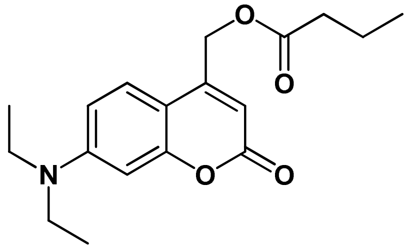

A solution of 50 mg (568 µmol) butyric acid and 20 mg DMAP (162 µmol) in 3 ml dry DCM was treated with a solution of 100 mg EDC (642 µmol) in 3 ml DCM and the reaction mixture was stirred for 5 min. A solution of 100 mg of hydroxycoumarin **15** (404 µmol) in 3 ml DCM was added subsequently and the reaction mixture stirred for additional 6 h. The reaction mixture was poured into a mixture of EtOAc and $H_2O$ (1:1, 200 ml) and the layers were separated. The organic layer was washed with $H_2O$ (50 ml), sat. NaCl solution (50 ml) and dried over $Na_2SO_4$. The solvent was removed under reduced pressure and the residue purified by flash chromatography (eluent cyclohexane : EtOAc 5:1). The title compound was obtained as yellowish oil (98 mg, 309 µmol, 76 %).

$^1$H-NMR (MeOD) δ = 7.47 (d, *J* = 9.0 Hz, 1H), 6.75 (dd, *J* = 2.6, 9.1 Hz, 1H), 6.55 (d, *J* = 2.6 Hz, 1H), 6.04 (s, 1H), 5.31 (s, 2H), 3.48 (q, *J* = 7.2 Hz, 4H), 2.46 (t, *J* = 7.3 Hz, 2H), 1.71 (m, *2*H), 1.21 (t, *J* = 7.4 Hz, 6H), 0.99 (t, *J* = 7.4 Hz, 3H) ppm.

$^{13}$C-NMR (MeOD) δ = 174.59, 164.60, 157.75, 153.21, 152.73, 126.40, 110.66, 107.38, 106.27, 98.49, 62.58, 45.81, 36.88, 19.61, 14.12, 12.90 ppm.

HR-MS (ESI positive) m/z found 318.17003, calculated for $C_{18}H_{24}NO_4$ 318.170534 $[M+H]^+$.

(7-*N*,*N*-Diethylamino)-2-oxo-2*H*-chromen-4-yl)methyl oleate (**10**)

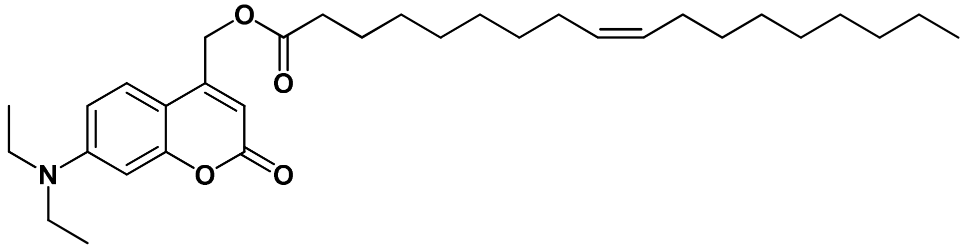

A solution of 200 mg (0.71 mmol) oleic acid and 20 mg DMAP (162 µmol) in 5 ml dry DCM was treated with a solution of 200 mg EDCxHCl (1.04 mmol) in 5 ml DCM and the reaction mixture was stirred for 5 min. A solution of 100 mg of hydroxycoumarin **15** (404 µmol) in 5 ml DCM was added subsequently and the reaction mixture stirred for additional 6 h. The reaction mixture was poured into a mixture of EtOAc and $H_2O$ (1:1, 200 ml), sat. NaCl solution (50 ml) was added and the layers were

separated. The organic layer was washed with $H_2O$ (100 ml), sat. NaCl solution (100 ml) and dried over $Na_2SO_4$. The solvent was removed under reduced pressure and the residue purified by flash chromatography (eluent cyclohexane : EtOAc 10:1). The title compound was obtained as yellowish oil (144 mg, 281 μmol, 76 %).

$^1$H-NMR (400 MHz, $CDCl_3$) δ = 7.28 (d, *J* = 8.8 Hz, 1H), 6.56 (dd, *J* = 9.0 Hz, 1H), 6.49 (s, 1H), 6.10 (s, 1H), 5.36-5.27 (m, 2H), 5.20 (s, 2H), 3.40 (q, *J* = 7.0 Hz, 4H), 2.42 (t, *J* = 7.5 Hz, 2H), 2.05-1.95 (m, 4H) 1.71-1.63 (m, 2H), 1.46-1.16 (m, 26H), 0.87 (t, *J* = 6.5 Hz, 3H) ppm.

$^{13}$C-NMR (101 MHz, $CDCl_3$) δ = 172.94, 161.74, 156.26, 150.65, 149.55, 129.97, 129.68, 124.40, 108.62, 106.45, 106.04, 97.80, 61.13, 44.71, 34.08, 31.88, 29.74, 29.65, 29.50, 29.29, 29.11, 29.10, 29.06, 27.20, 27.13, 24.85, 22.65, 14.07, 12.41 ppm.

HR-MS (ESI positive) m/z found 534.35647, calculated for $C_{32}H_{49}NO_4Na$ 534.355929 $[M+H]^+$.

(7-*N*,*N*-Di(tert-butoxycarbonylmethyl)amino)-2-oxo-2*H*-chromen-4-yl)methyl arachidonate **17**

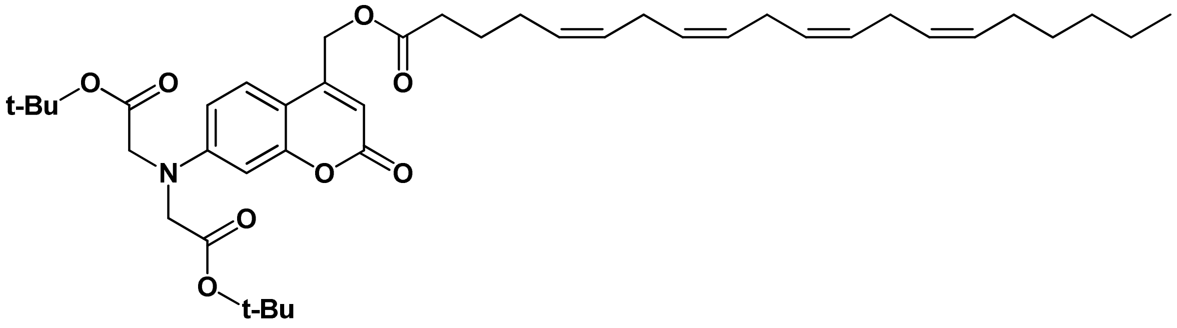


A solution of 100 mg (329 μmol) arachidonic acid and 50 mg DMAP (405 μmol) in 3 ml dry DCM was treated with a solution of 100 mg EDC (642 μmol) in 3 ml DCM and the reaction mixture was stirred for 5 min. A solution of 150 mg of the *tert*-butyl protected hydroxycoumarin **16** (358 μmol) in 3 ml DCM was added subsequently and the reaction mixture stirred for additional 6 h. The reaction mixture was poured into a mixture of EtOAc and $H_2O$ (1:1, 200 ml) and the layers were separated. The organic layer was washed with $H_2O$ (50 ml), sat. NaCl solution (50 ml) and dried over $Na_2SO_4$. The solvent was removed under reduced pressure and the residue purified by flash chromatography (eluent cyclohexane : EtOAc 10:1). The title compound was obtained as yellowish oil (123 mg, 174 μmol, 53 %).

$^1$H-NMR (MeOD) δ = 7.47 (d, *J* = 9.0 Hz, 1H), 6.59 (dd, *J* =2.6, 9.0 Hz, 1H), 6.44 (s, 1H), 6.12 (s, 1H), 5.39 - 5.27 (m, 8H), 4.16 (s, 4H), 2.80 (m, 6H), 2.47 (t, *J* = 7.3 Hz, 2H), 2.14 (q, *J* = 6.8 Hz, 2H), 2.03 (q, *J* = 6.8 Hz, 2H), 1.73 (m, 2H), 1.48 (s, 18H), 1.20 - 1.39 (m, 8H), 0.88 (t, *J* = 6.8 Hz, 3H) ppm.

$^{13}$C-NMR (MeOD) δ = 174.30, 170.95, 163.62, 156.95, 153.20, 152.46, 131.38, 130.27, 130.04, 129.63, 129.33, 129.09, 128.95, 126.32, 110.89, 109.25, 108.46, 99.99, 83.41, 62.53 ppm.

HR-MS (ESI positive) m/z found 706.43477, calculated for $C_{42}H_{60}NO_8$ 706.431894 $[M+H]^+$.

(7-*N*,*N*-Di(carboxymethyl)amino)-2-oxo-2*H*-chromen-4-yl)methyl arachidonate **7**

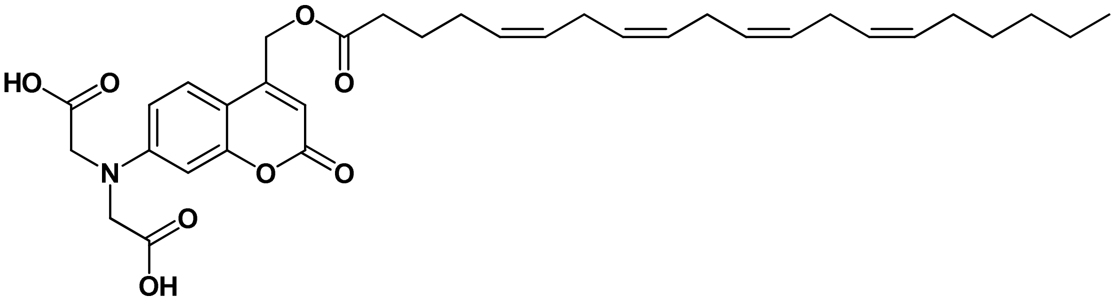

60 mg of **17** (85 μmol) were placed in a cooled pear-shaped flask equipped with a stirring bar. A mixture of 3 ml TFA and 1 ml DCM was added under vigorous stirring. The reaction mixture was stirred for an additional 15 min at 0 °C and subsequently poured into a mixture of EtOAc and $H_2O$. The layers were separated and the organic layer washed with saturated NaCl solution (100 ml) and dried over $Na_2SO_4$. The solvent was removed under reduced pressure and the residue subjected to isocratic HPLC purification (93 % MeOH, 7 % $H_2O$, 0.04 % TFA, 10 ml/min on a 250x10 mm LiChrospher 100 RP-18 column). The title compound was obtained as a yellowish oil (28 mg, 47 μmol, 55%)

$^1$H NMR (400 MHz, $CDCl_3$) δ = 8.46 ($s_{br}$, 2H), 7.19 (s, 1H), 6.45-6.27 (m, 2H), 6.05 (s, 1H), 5.44 – 5.19 (m, 8H), 5.07 (s, 2H), 4.17 (s, 4H), 2.82 – 2.64 (m, 6H), 2.38 (t, *J*=7.5, 2H), 2.06 (dd, *J*=13.7, 6.9 Hz, 2H), 1.97 (dd, *J*=6.9 Hz, 2H), 1.75 – 1.60 (m, 2H), 1.41 – 1.10 (m, 3H), 0.80 (t, *J*=6.7, 8H) ppm.

$^{13}$C NMR (100 MHz, $CDCl_3$) δ = 174.46, 173.07, 162.38, 155.09, 150.55, 149.72, 130.52, 129.18, 128.64, 128.62, 128.35, 128.05, 127.83, 127.53, 125.01, 109.28, 108.60, 107.69, 99.09, 60.91, 54.75, 33.40, 31.52, 29.32, 27.22, 26.50, 25.64, 24.64, 22.58, 14.09 ppm.

$^1$H resonances of the coumarin part of the spectrum are very broad; the corresponding $^{13}$C signals are significantly less intense, probably due to two conformations in equilibrium and/or metal coordination.

MS (ESI negative) m/z found 692.29038 $[M-H]^-$, calculated for $C_{34}H_{46}NO_{10}S_2^-$ 692.25631.

# $^{1}H$ and $^{13}C$ NMR spectra of new compounds

## $^{1}H$-NMR spectrum of **1**

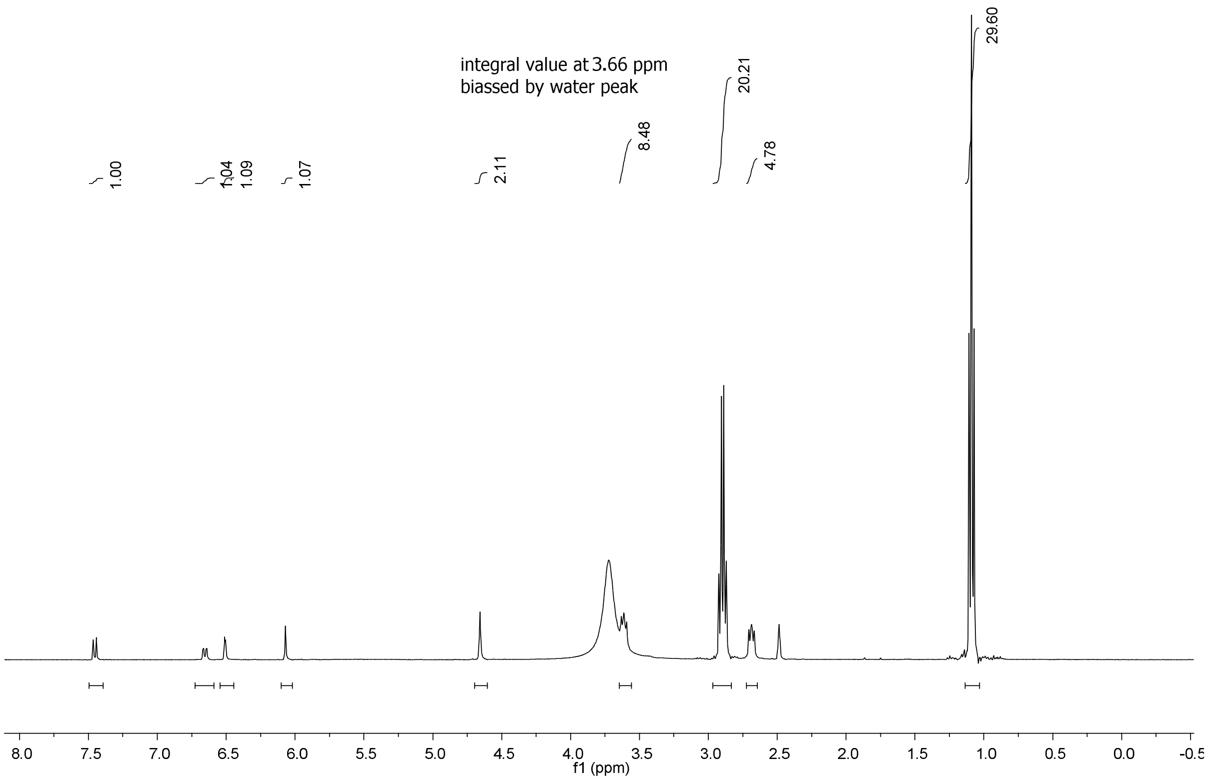

<sup></sup>

$^{13}$C-NMR spectrum of **1**

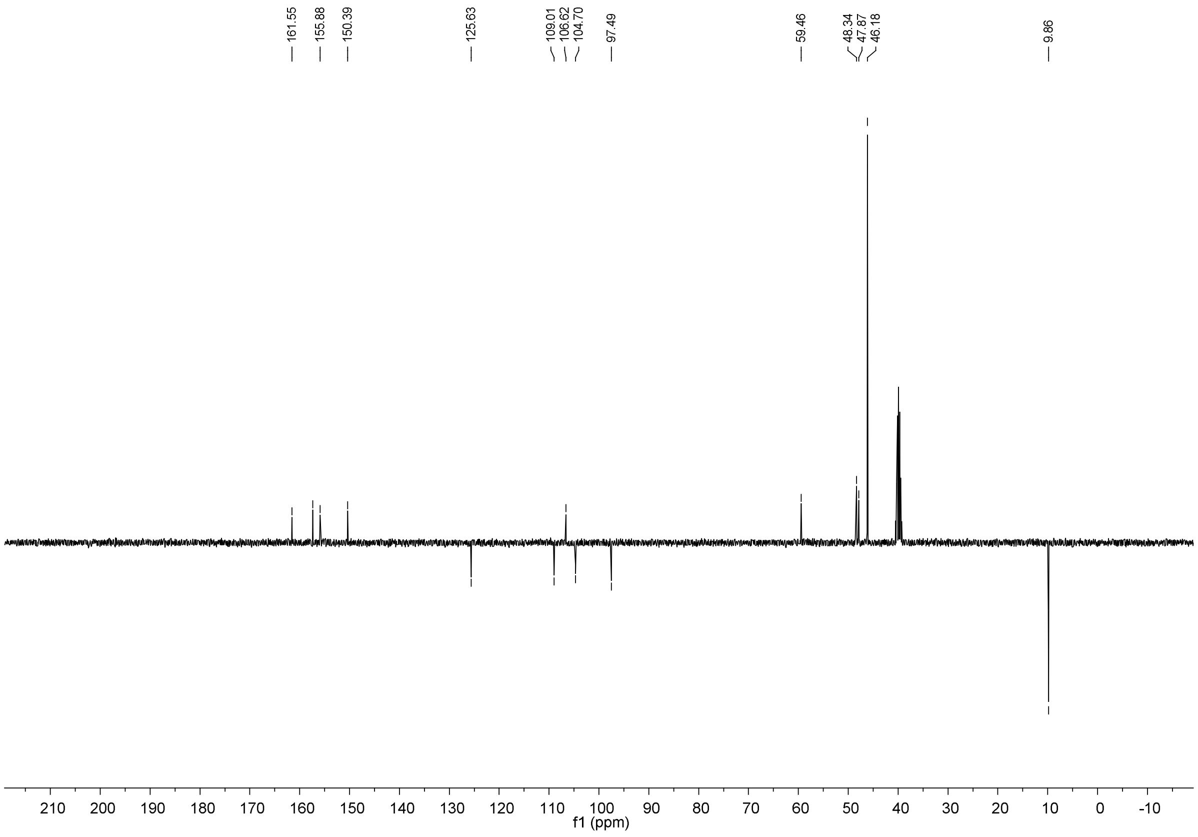


$^{1}$H-NMR spectrum of **4**

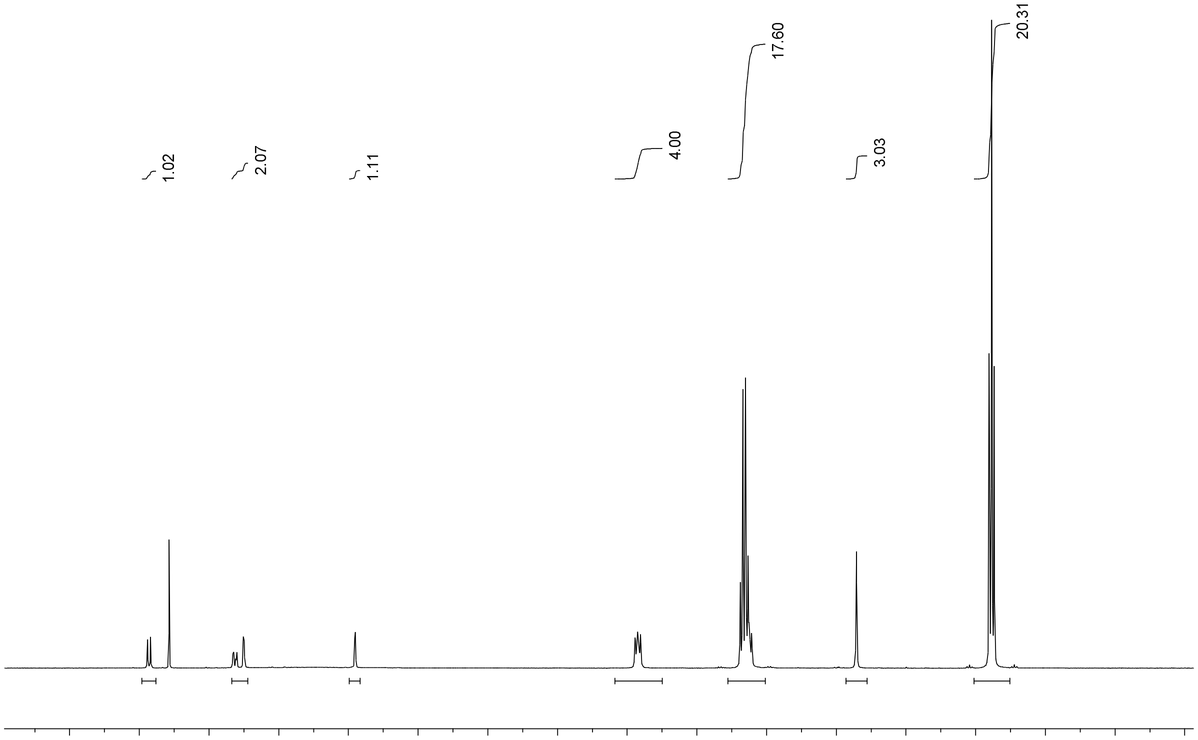

$^{13}$C-NMR spectrum of **4**

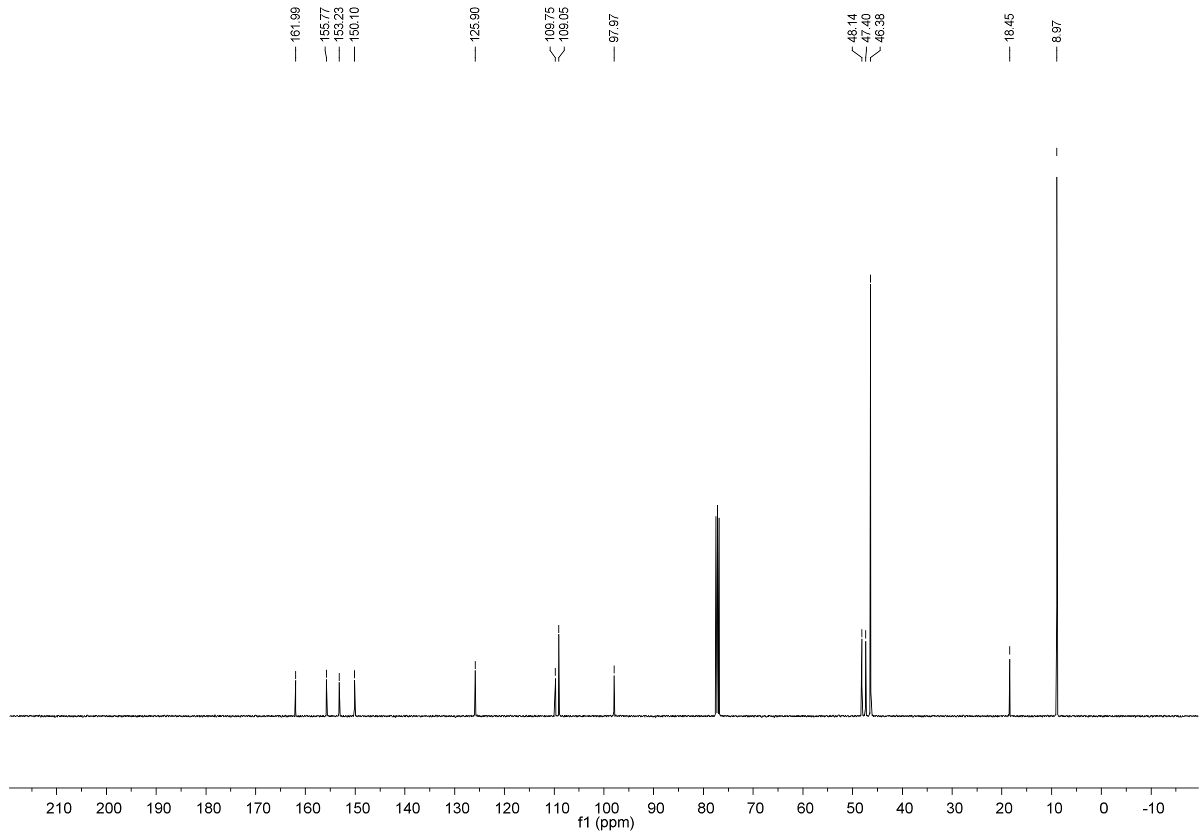


$^{1}$H-NMR spectrum of **5**

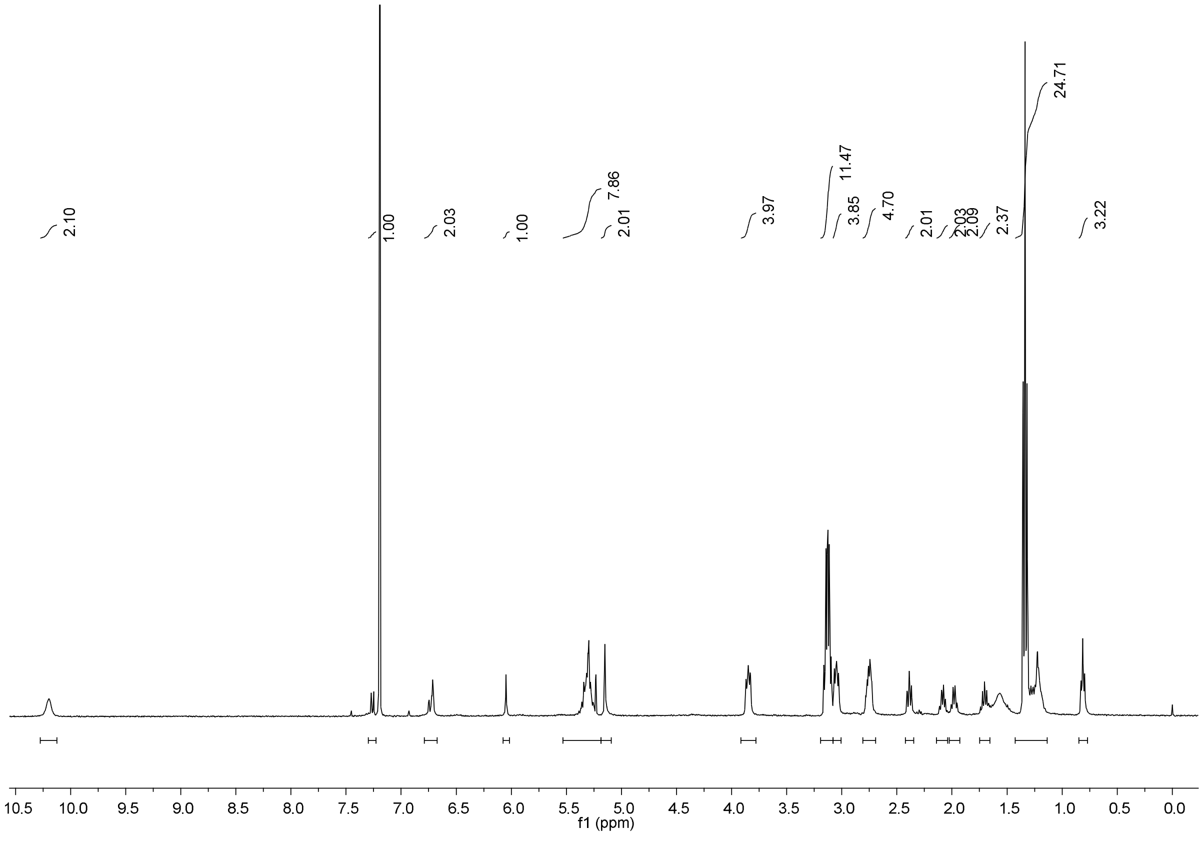

$^{13}$C-NMR spectrum of **5**

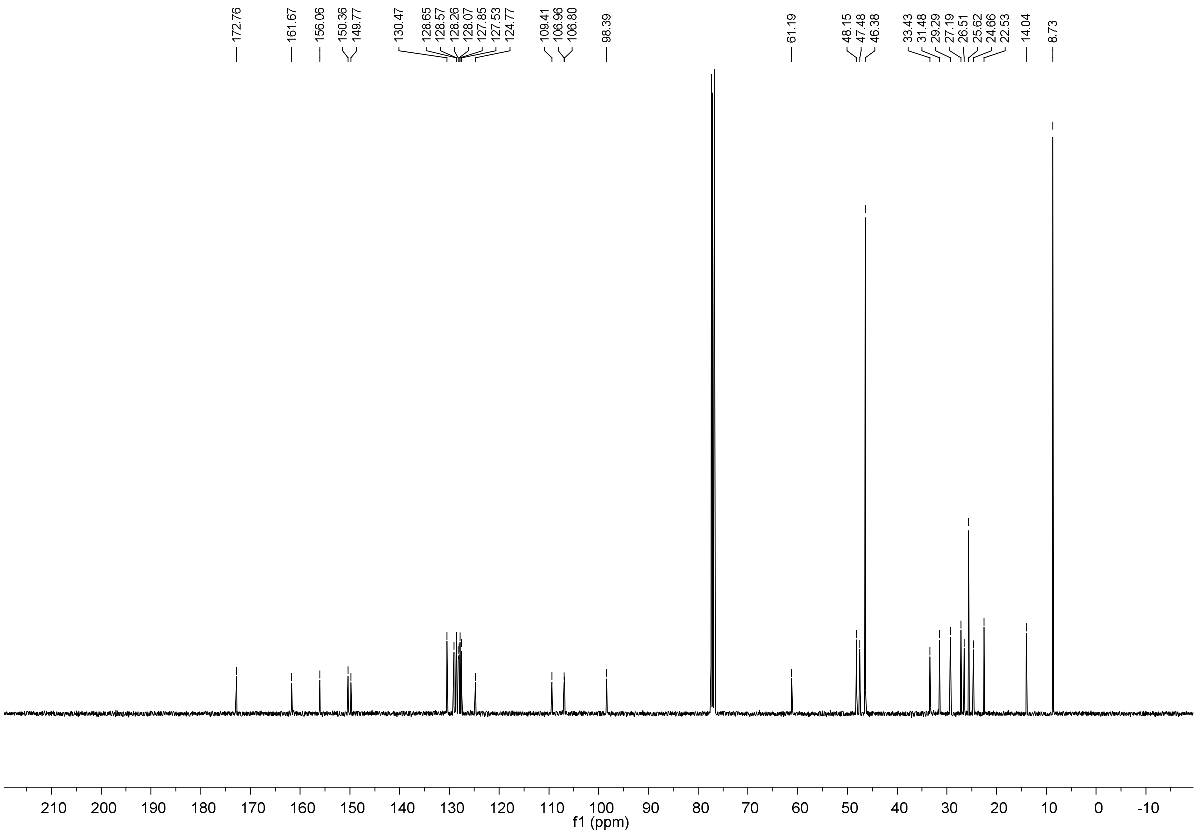


$^{1}$H-NMR spectrum of **7**

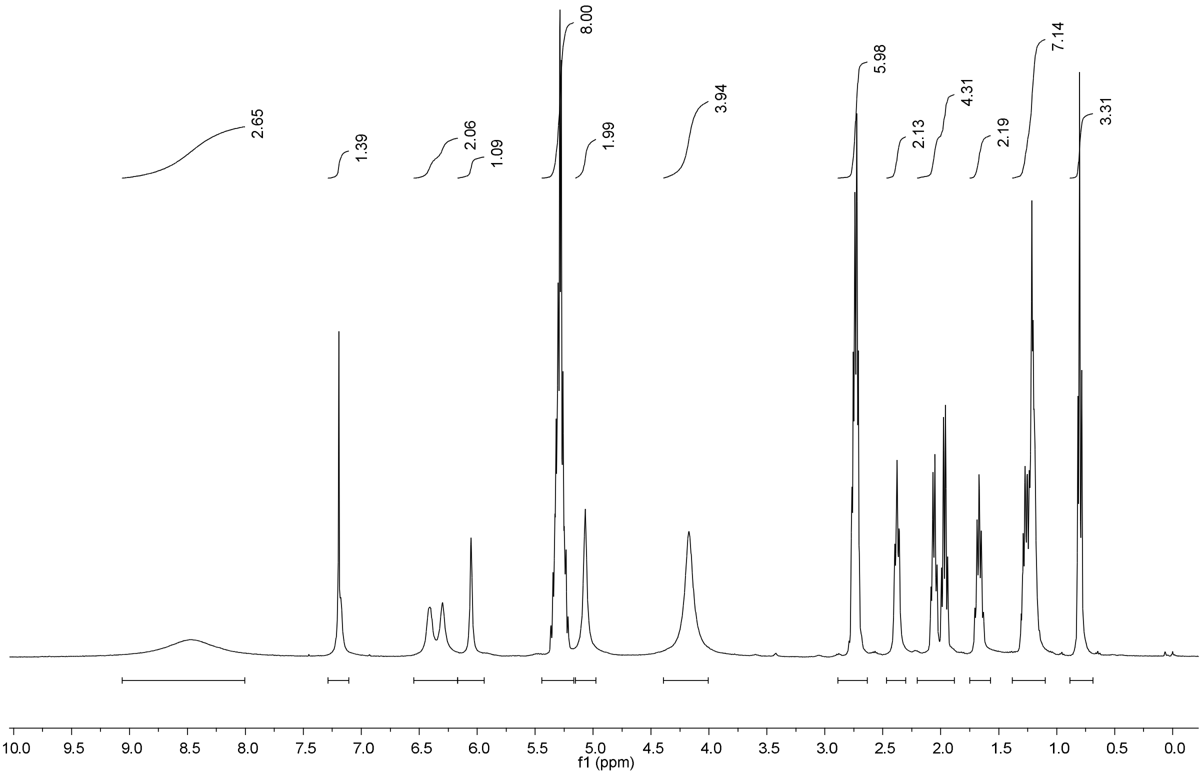

$^{13}$C-NMR spectrum of **7**

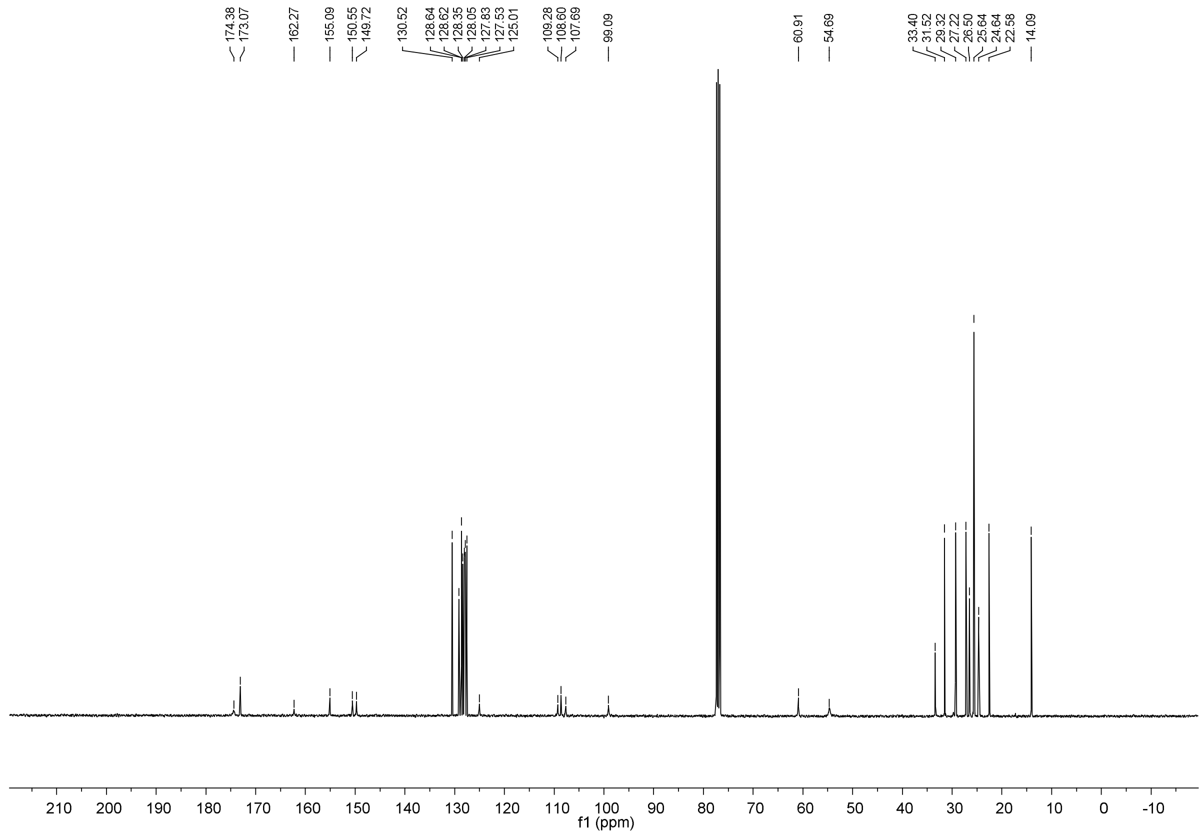


$^{1}$H-NMR spectrum of **8**

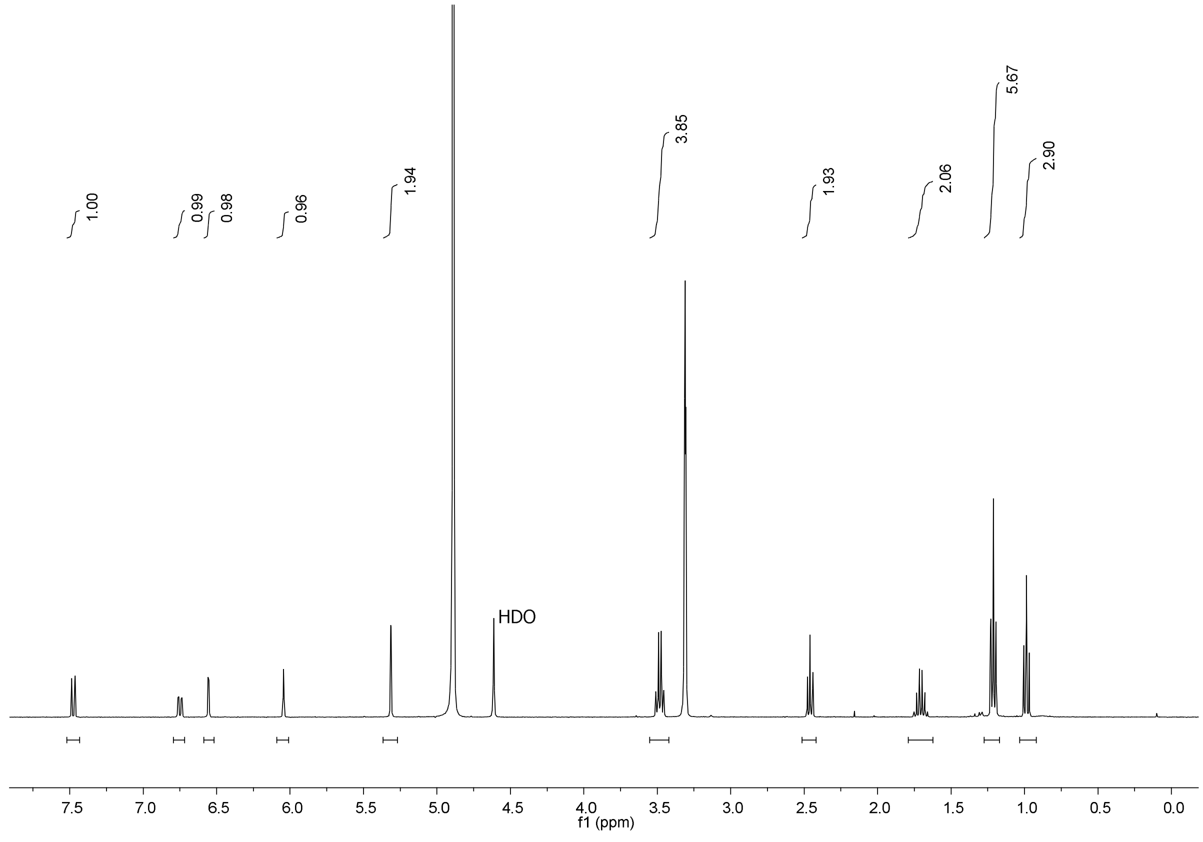

$^{13}$C-NMR spectrum of **8**

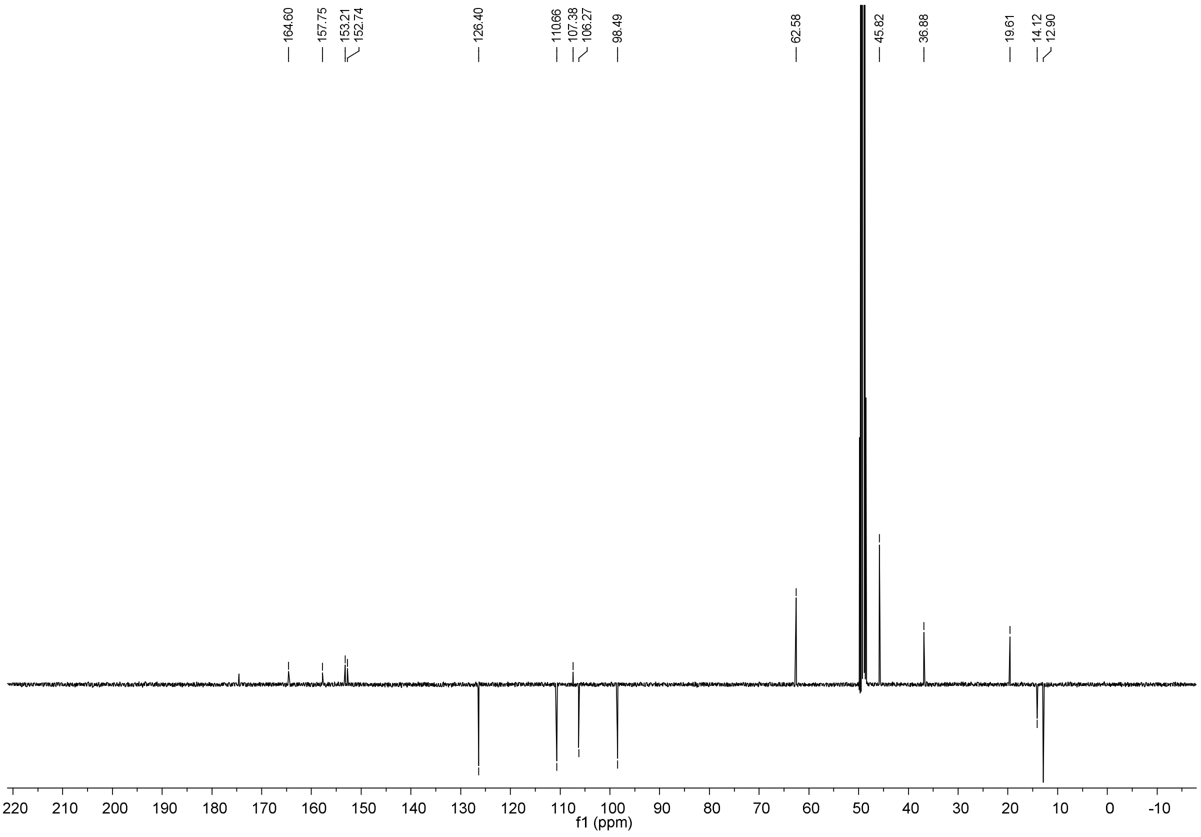


$^{1}$H-NMR spectrum of **9**

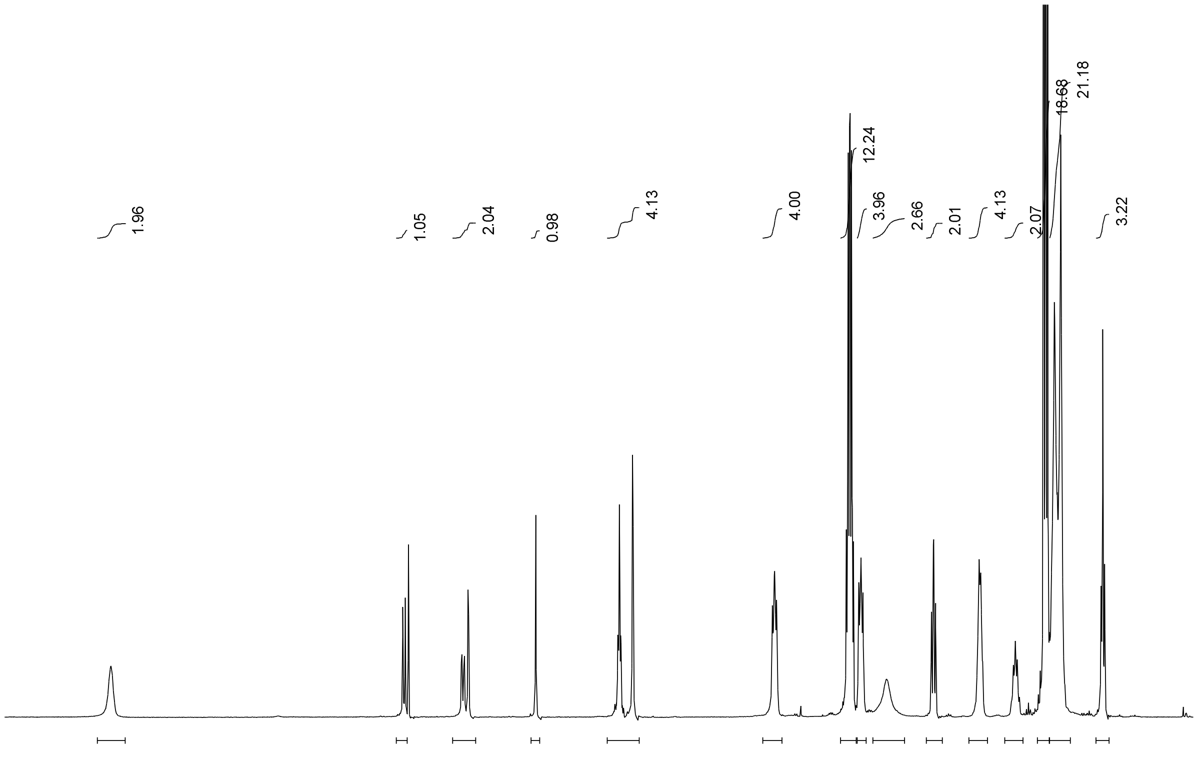

$^{13}$C-NMR spectrum of **9**

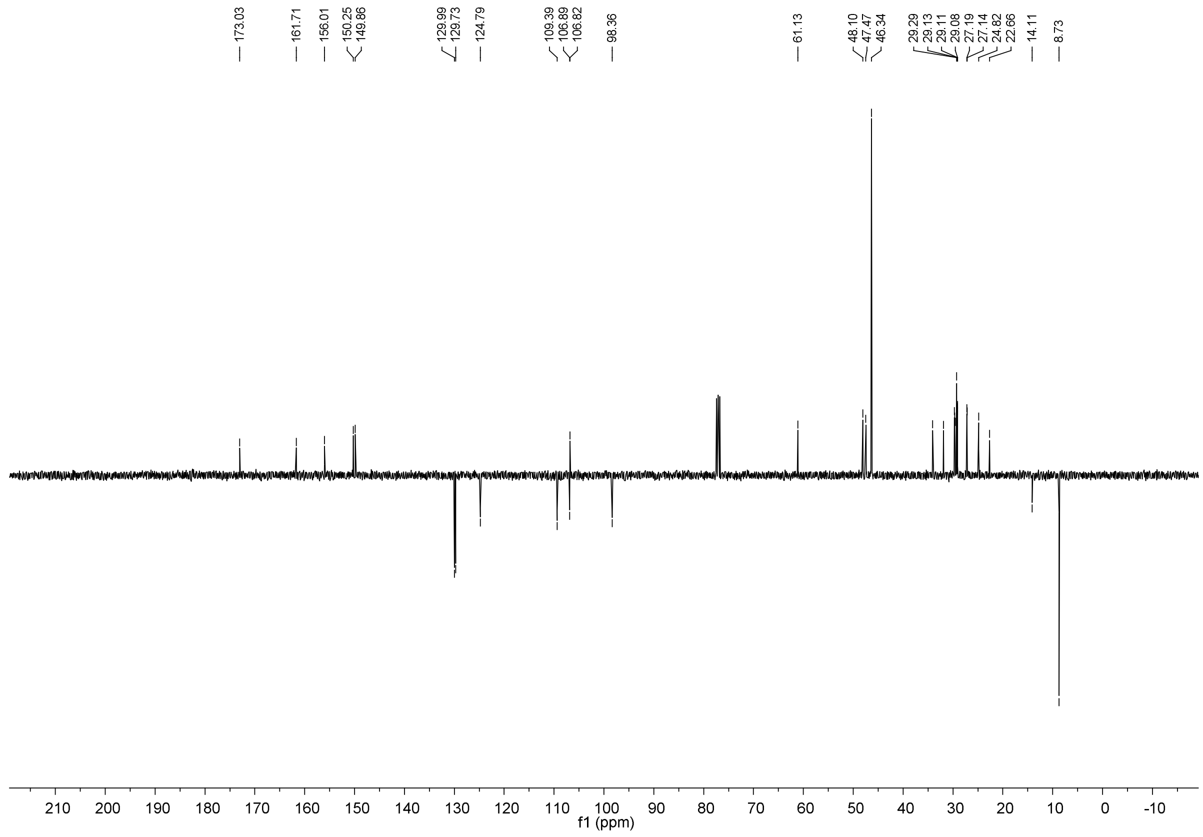


$^{1}$H-NMR spectrum of **10**

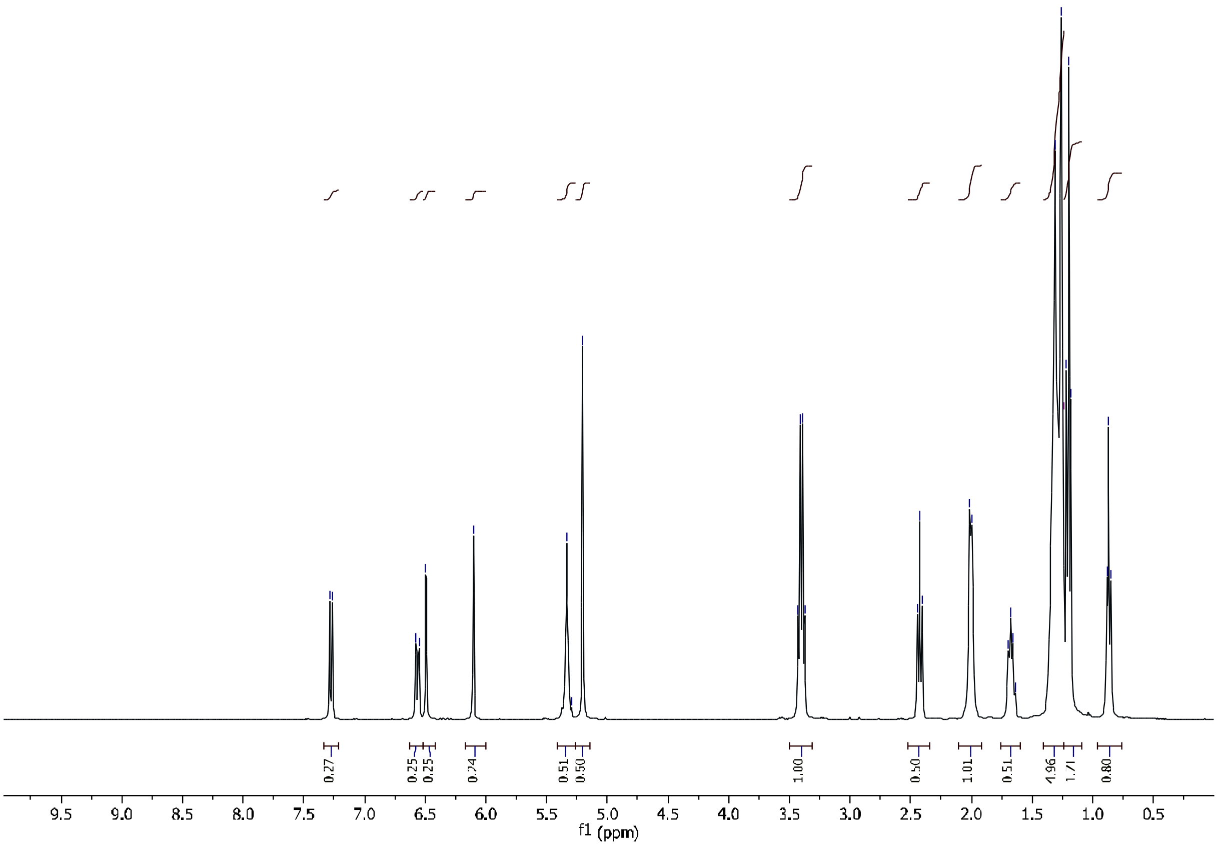

$^{13}$C-NMR spectrum of **10**

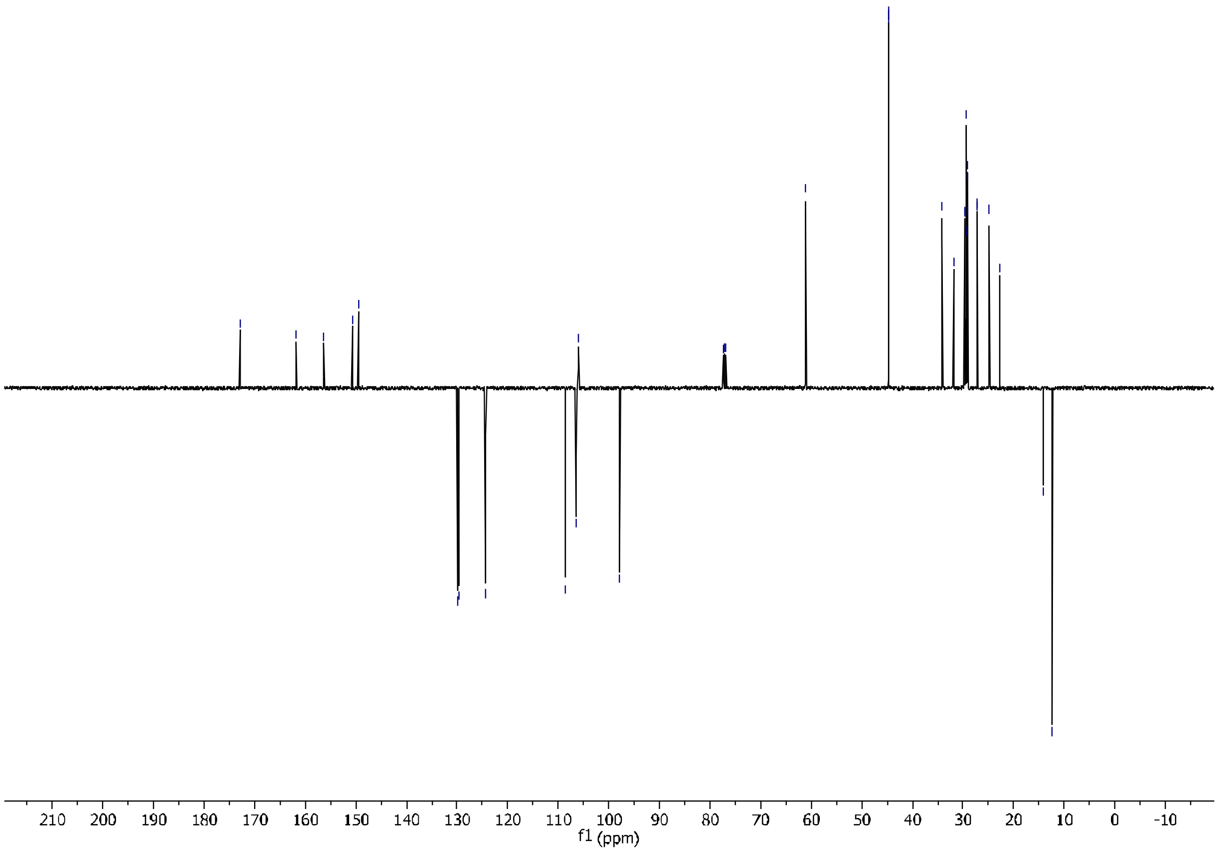


$^{1}$H-NMR spectrum of **17**

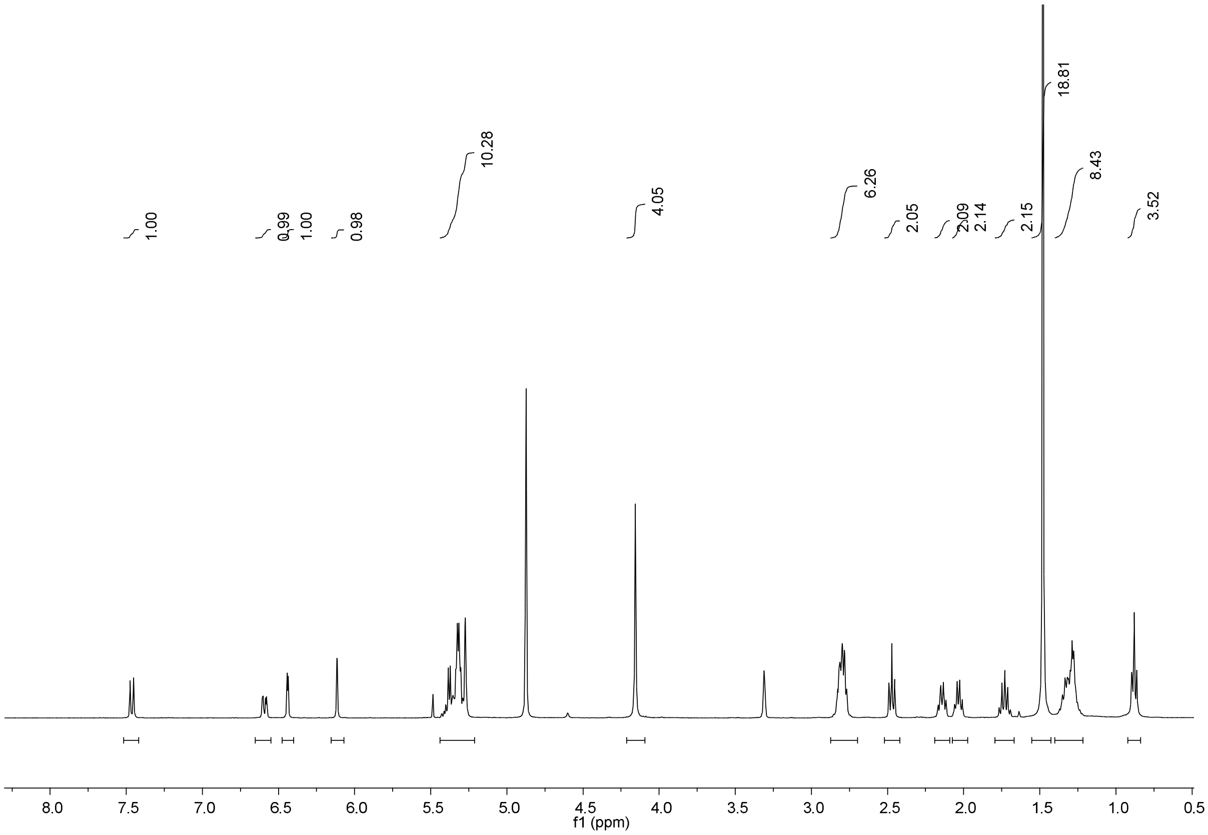

$^{13}$C-NMR spectrum of **17**

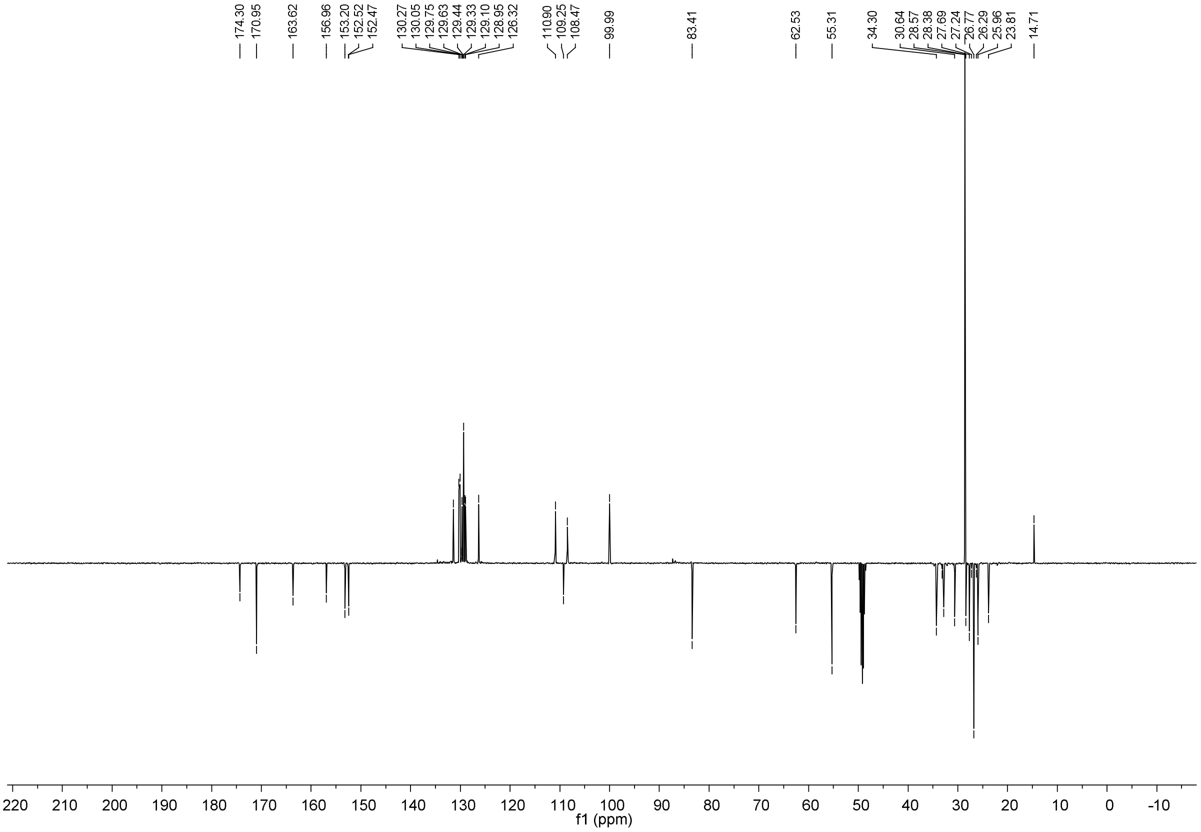

# Supplementary Methods

Image analysis, data processing and description of ImageJ macros.

<u>Quantification of photoreactions in living cells (main text, Fig. 2)</u>

Images were analyzed using Fiji and the newly developed ImageJ macro called “PM/background ratio-calculator Macro” (see “Overview of the image analysis pipeline” on page 33 for the general pipeline). Raw images were loaded into Fiji and the macro was started to perform all image analysis steps including cell segmentation, classification of plasma membrane and background pixels, ratio calculation and feature extraction. Mean pixel intensities for each cell and channel are summarized in a data table and multiple time-trace-plots.
The cell segmentation procedure is described on page 34 and is critical for the image analysis. Therefore, user-interference is needed to roughly define the cell outlines with the ImageJ’s “Pencil Tool”. The defined regions of interestes (ROIs) are further used to classify each pixel in each ROI in plasma membrane (PM) and background (intracellular space / vesicles). It was assumed that the plasma membrane is located at the outer rim of each ROI (see page 35 for details). The mean intensity of the PM and the background was computed for each ROI after pixel classification and a ratio of both values was computed with single cell resolution.
All single-cell time-traces were normalized by dividing each time-point with the average of time-points prior to uncaging. Then, normalized data were used to compute averaged time-traces including standard deviation and standard error values by computing the mean over all single-cell time-traces for each time-point.

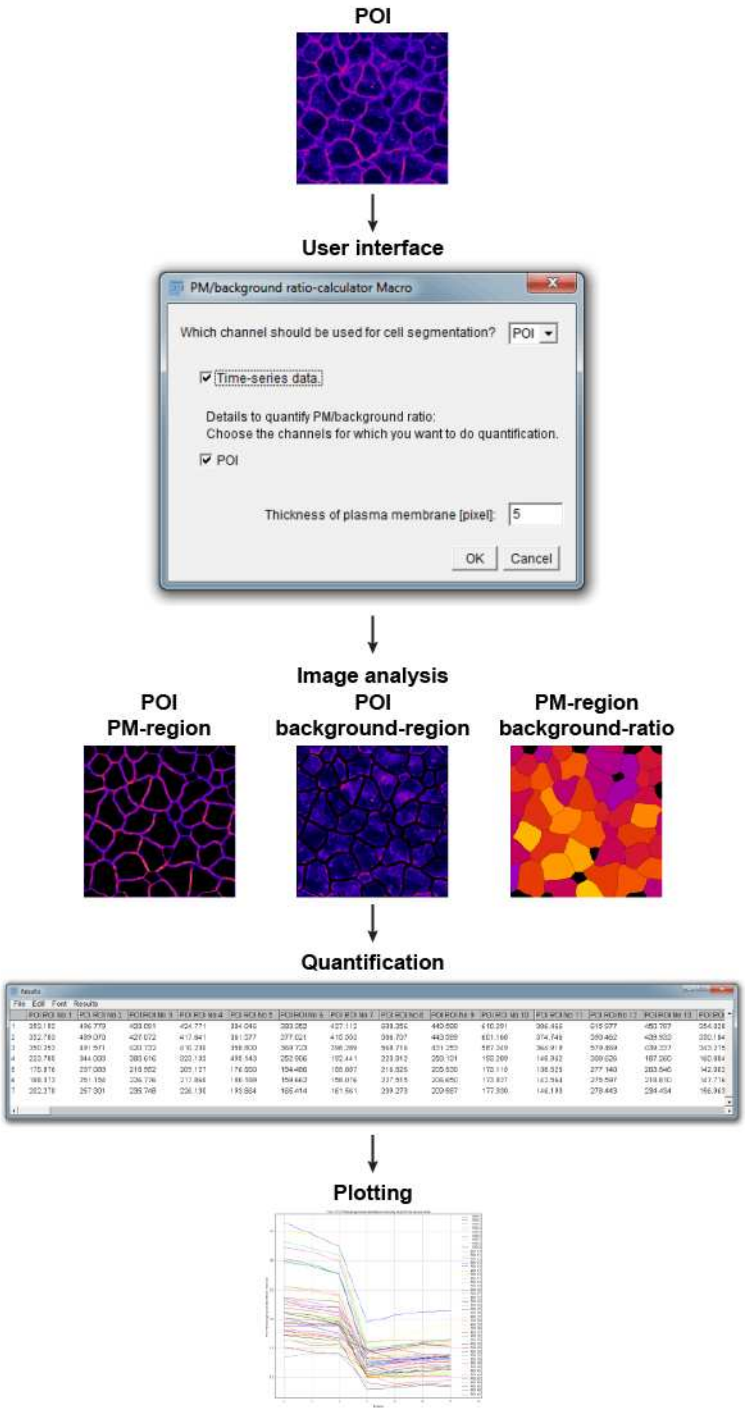


Overview of the image analysis pipeline of "PM/background ratio-calculator Macro". First images are loaded into Fiji and the macro is started. The graphical user interface lets the user define a few parameters such as the plasma membrane width. The macro facilitates cell segmentation, pixel classification and feature extraction and summarizes the results in a data table and multiple plots.

| **Image processing step:** | **sample image** | **ImageJ/FIJI command:** |
|---|---|---|
| 1.) Modification of cell segmentation channel | 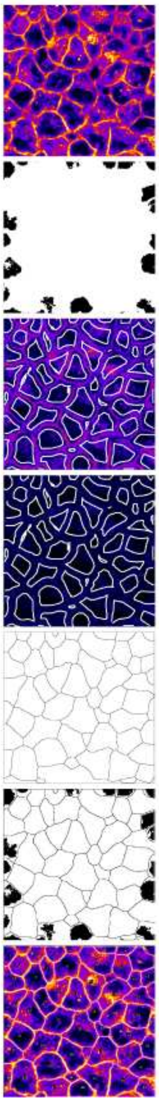 | 1a) Image>Duplicate<br>1b) Image>Type>8-bit<br>1c) Image>Stacks>Z Project...<br>Select: 'Average Intensity', 'OK'<br>d) Process>Filters>Median<br>Select Radius = 1 pixel |
| 2.) Create binary mask | | 2.) Image>Adjust>Threshold...<br>Adjust treshold to your needs...<br>Select: 'Dark Background', 'Auto', 'Apply'<br>Process>Filters>Median - 1 pixel radius<br>Process>Binary>Open |
| 4.) Create manual cell mask and use 'voronoi' algorithm | | 4a) Select 'Pencil Tool'<br>Use Pencil Width of at least 5 pixels.<br>Pick Foreground color = 255<br>Outline cells with 'Pencil Tool' |
| | | 4b) Threshold cell outlines:<br>Image>Adjust>Threshold...<br>Set to: 254 to 255; 'Apply' |
| | | 4c) 'Voronoi' algorithm:<br>Process>Binary>Voronoi<br>Image>Adjust>Threshold...<br>Set to: 1 to 255; 'Apply'<br>Edit>Invert<br>Process>Math>Subtract...<br>Select: Value = 255; 'OK' |
| | | 4d) Process>Image Calculator...<br>Select: Binary Mask and Voronoi Mask<br>Operation 'Multiply'; 'OK' |
| 5.) Analyze particles and add them to the ROI Manager | | 5.) Analyze>Analyze Particles...<br>Select: 'Add to Manager', 'OK' |

Pipeline for cell segmentation. First, a thresholding approach is chosen to distinguish cells from background and the user roughly draws the cell outlines afterwards in order to define cell locations with the 'voronoi'-algorithm.

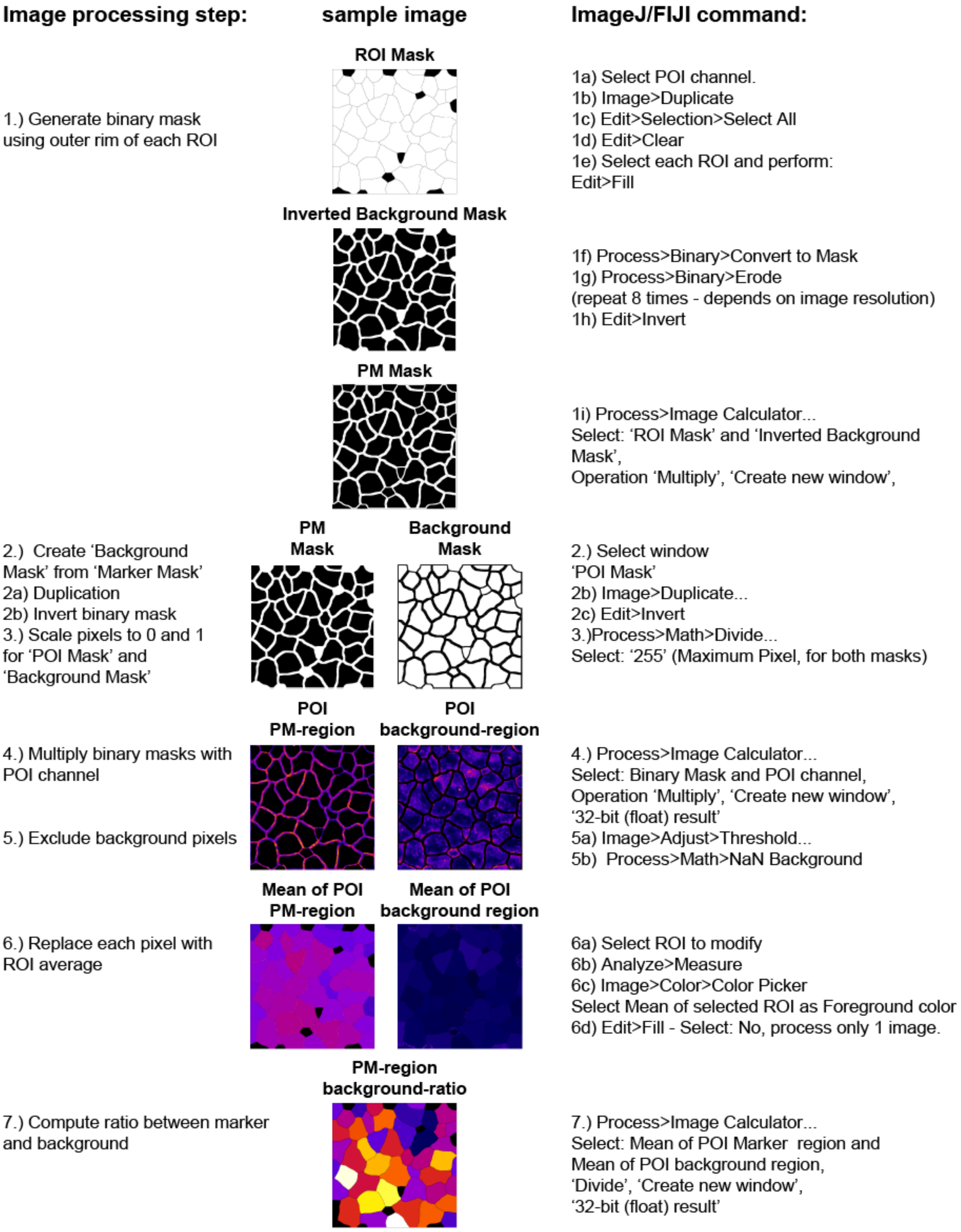


Pipeline for computing PM-region background-ratios. ROIs are used to define the PM location by computing the outer rim of each ROI. This information is further used to i) classify pixels into PM-region and backgrond-region and ii) computing its mean and calculating the PM-region background-ratio.

Analysis of calcium oscillations

Single cell traces of $Ca^{2+}$ oscillations were obtained by automatized analysis using the ImageJ-based macro FluoQ. FluoQ performed all steps in the image analysis and summarized the results in multiple graphical and numerical displays. It also saved all measured parameters in a file named "EXPNAME_dataset.txt" ("EXPNAME" is a placeholder for the user given name of the experiments).

A reference for FluoQ is given in the main text, and the macro may be downloaded from the *ACS Chemical Biology* homepage (http://pubs.acs.org/doi/abs/10.1021/cb4003442). The output file was loaded into R in order to perform the downstream data analysis and extract the intracellular calcium dynamics. The R script is provided as .R file alongside the supporting information. For the peak detection, the R package "Peaks" (reference given in the main text) was employed and the "SpectrumSearch"-function was called (arguments: background = F, markov = F, threshold = 5, sigma = 2). In order to evaluate the significance of each identified peak, a running z-score ($z_t$) was calculated according to the following equation ($\tilde{x}_{0.5} = median,\ sd = standard\ deviation$):

$$x_t^{med} = median([x_{t-1}, x_{t+1}]) \qquad \text{(III)}$$

$$x_t^{adj} = x_t - \min([x_{t-10}^{med}, x_{t+10}^{med}]) \qquad \text{(IV)}$$

$$z_t = \frac{x_t^{adj}}{sd\left(\left\{x^{adj} | x^{adj} \leq \tilde{x}_{0.5}^{adj}\right\}\right)} \qquad \text{(V)}$$

First, a running median was calculated for each time-trace ($x_t^{med}$), which was used to calculate a running minimum. This estimate was subtracted from the time-trace ($x_t^{adj}$) and used to calculate a the z-score ($z_t$). In order to estimate the standard deviation ($sd$) of each time-trace, only values of $x_t^{adj}$ below or equal the median were taken into account ($\left\{x^{adj} | x^{adj} \leq \tilde{x}_{0.5}^{adj}\right\}$). Thereby, only values between the calcium peaks were used to estimate the standard deviation and high intensity peaks were excluded. In the following, only identified peaks ($Peaks$) that exhibited a running z-score ($z_t$) above 3 were used for further downstream statistics. The $ratio$ describes the fraction of the longest observed period over the second longest observed period.

$$Peaks(x_t) = \{Peaks | z_{Peaks} > 3\} \qquad \text{(VI)}$$

$$sum(Peaks(x_t)) = \sum_{i=1}^{n} p_i \qquad \text{(VII)}$$

Furthermore, each calcium time-trace was classified into one of the five response patterns. For this, each time-trace was split into three different parts ($st$ is the time of uncaging or stimulation), their corresponding averages ($\bar{x}$) and changes ($\Delta$) were determined.

$$\bar{x}_{baseline} = \frac{1}{st} \sum_{i=1}^{st} x_i \qquad \text{(VIII)}$$

$$\bar{x}_{immediate\ response} = \frac{1}{372} \sum_{i=st+30}^{st+402} x_i \qquad \text{(IX)}$$

$$\bar{x}_{late\ response} = \frac{1}{n-(st+402)} \sum_{i=st+402}^{n} x_i \qquad \text{(X)}$$

$$\bar{x}_{total\ response} = \frac{1}{n-(st-30)} \sum_{i=st+30}^{n} x_i \qquad \text{(XI)}$$

$$\Delta_{immediate} = \frac{\bar{x}_{baseline} - \bar{x}_{immediate\ response}}{\bar{x}_{baseline}} \qquad \text{(XII)}$$

$$\Delta_{late} = \frac{\bar{x}_{immediate\ response} - \bar{x}_{late\ response}}{\bar{x}_{immediate\ response}} \qquad \text{(XIV)}$$

$$\Delta_{total} = \frac{\bar{x}_{baseline} - \bar{x}_{total\ response}}{\bar{x}_{baseline}} \qquad \text{(XV)}$$

Individual cell traces were then classified according to the following scheme:

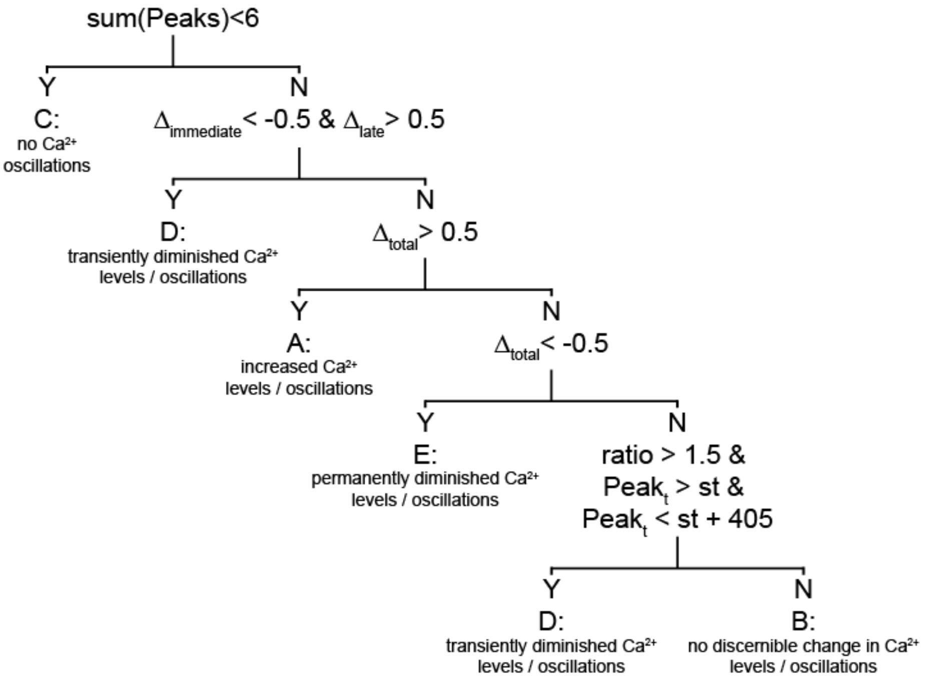

Supplement: Supplementary Information — Supplementary Figures 1-16, Supplementary Note 1 and Supplementary Methods [file ncomms10056-s1.pdf]
